# Workflows for microarray data processing in the Kepler environment

**DOI:** 10.1186/1471-2105-13-102

**Published:** 2012-05-17

**Authors:** Thomas Stropp, Timothy McPhillips, Bertram Ludäscher, Mark Bieda

**Affiliations:** 1 Department of Biochemistry and Molecular Biology, University of Calgary, Calgary, AB, Canada; 2 Genome Center, University of California-Davis, Davis, CA, USA

## Abstract

**Background:**

Microarray data analysis has been the subject of extensive and ongoing pipeline development due to its complexity, the availability of several options at each analysis step, and the development of new analysis demands, including integration with new data sources. Bioinformatics pipelines are usually custom built for different applications, making them typically difficult to modify, extend and repurpose. Scientific workflow systems are intended to address these issues by providing general-purpose frameworks in which to develop and execute such pipelines. The Kepler workflow environment is a well-established system under continual development that is employed in several areas of scientific research. Kepler provides a flexible graphical interface, featuring clear display of parameter values, for design and modification of workflows. It has capabilities for developing novel computational components in the R, Python, and Java programming languages, all of which are widely used for bioinformatics algorithm development, along with capabilities for invoking external applications and using web services.

**Results:**

We developed a series of fully functional bioinformatics pipelines addressing common tasks in microarray processing in the Kepler workflow environment. These pipelines consist of a set of tools for GFF file processing of NimbleGen chromatin immunoprecipitation on microarray (ChIP-chip) datasets and more comprehensive workflows for Affymetrix gene expression microarray bioinformatics and basic primer design for PCR experiments, which are often used to validate microarray results. Although functional in themselves, these workflows can be easily customized, extended, or repurposed to match the needs of specific projects and are designed to be a toolkit and starting point for specific applications. These workflows illustrate a workflow programming paradigm focusing on local resources (programs and data) and therefore are close to traditional shell scripting or R/BioConductor scripting approaches to pipeline design. Finally, we suggest that microarray data processing task workflows may provide a basis for future example-based comparison of different workflow systems.

**Conclusions:**

We provide a set of tools and complete workflows for microarray data analysis in the Kepler environment, which has the advantages of offering graphical, clear display of conceptual steps and parameters and the ability to easily integrate other resources such as remote data and web services.

## Background

There have been dimensional increases in ‘omics datasets, including introduction of new types of data, increases in the size of individual datasets, increases in the varieties of experimental platforms within a given data type (e.g. more varieties of gene expression microarrays), and very large increases in the total number of datasets being generated. This explosion of varieties and amount of data has led to a large increase in the number of bioinformatics tools; R/BioConductor has grown from 123 packages in 2006 to 517 as of 2011 [1]. The increasing number of types of datasets has led to a large increase in the demands placed on analyses, with new needs for cross dataset comparisons (e.g. [2,3]) and correlations between different platforms [4]. In total, this has led to a highly dynamic and rapidly changing analysis environment, with increasingly complicated and non-standard data analysis needs. Under these conditions, systems that can be rapidly modified, extended, and yet easily understood are highly valued.

It has long been recognized that development of bioinformatics pipelines is a good way to organize complex analyses [5]. To ease creation of bioinformatics pipelines, the bioinformatics community has developed several large projects that have produced high-quality, often well-tested prewritten components for pipeline development (e.g. BioPerl [6], R/BioConductor [7]). In addition, for highly used microarray analysis tasks (e.g. Affymetrix gene expression microarray analysis), there has also been limited, but growing, production of fully developed analysis pipelines (e.g. [8-10]), sometimes hosted via websites [10,11]. However, it appears that most typical laboratory pipelines are developed in an ad-hoc manner, often using shell scripting or similar approaches [5]. For complex pipelines, these approaches often yield pipelines that are difficult to understand, modify or extend.

Analysis of large, complex datasets involving a large number of individual analysis steps is a general problem in many areas of science. To address these general needs, there has been both theoretical work and systems development in the computer science discipline of scientific workflow automation [12]. A workflow corresponds to a bioinformatics pipeline. Scientific workflow systems are developed with consideration of general pipeline needs, including a need for clarity of operations, appropriate level of abstraction of components, reusability, and easy extension and modification [13]. There are a number of scientific workflow systems that are applicable to bioinformatics pipelines, including Taverna [14], Loni Pipeline [15], Galaxy [11], Bioclipse [16], jORCA [17], and the system used in this work, Kepler [13]. Comparisons of the properties of these systems are presented in the original reports on the systems and more generally in [12,15]. We chose Kepler because it is a well-established, maintained system based on fundamental computer science principles and features a flexible, convenient graphical interface, an ability to clearly display parameters and comments on the workflow canvas, and the built-in presence of R support.

Microarrays are widely used in biological research for both gene expression and chromatin immunoprecipitation experiments (ChIP-chip). Microarray data analysis is complex and consists of several distinct steps with each step having a variety of options. There has been development of a large number of analysis approaches for microarray datasets and production of tools for individual steps in analysis [18]. Furthermore, there has been full pipeline development both in an open-source environment and commercial systems (e.g. [4,9,19]). The complexity and probable need for future extensibility (e.g. for linking to new gene and pathway information sources) of these pipelines indicate that workflow implementations are advantageous for microarray analysis systems.

The primary goal of this work is to provide fully functional workflows for critical microarray tasks in the Kepler environment, a well-supported workflow system used in other areas of scientific research. Although functional in themselves, these workflows are designed as a toolkit to be customized, extended, and repurposed for specific microarray analysis tasks. This toolkit is aimed at bioinformaticians interested in using scientific workflow systems for developing microarray analysis workflows for their specific applications. In particular, many of the workflow components (“actors” in Kepler terms) can be easily used for other purposes and Kepler can have actors that embody complete workflows. Effective use of this toolkit requires understanding of microarray analysis and, in many cases, R programming. However, these tools can be easily assembled into custom Kepler workflows that are designed for usage by “naive” end-users with little understanding of microarray analysis issues. We develop a series of utilities that are appropriate for basic analysis of ChIP-chip data in GFF format, which is the supplied format for Nimblegen Inc microarrays. For Affymetrix gene expression microarrays, we developed two closely related workflows embodying modified versions of a previously developed text-based pipeline [9]. PCR experiments are employed subsequent to ChIP-chip or gene expression microarray experiments for data validation. We present a full PCR primer design workflow using standard tools that can design primers around a user chosen region of any genome in the UCSC browser and subsequently displays the primer locations graphically. All of these workflows can serve as templates to enable further bioinformatics workflow development in the Kepler system. Our secondary goal was to demonstrate a programming paradigm based primarily on use of local resources (data and programs) as opposed to primary usage of remote web services. This paradigm is close to the oft-used scripting approach toward pipeline development. For example, our gene expression workflow demonstrates the translation of a fully local R-based pipeline into a workflow model. For gene expression microarray processing, the advantages of the workflow model over a traditional pipeline include clear abstraction of different conceptual stages and clear display of parameter values with graphical/textual categorization of different parameters. Finally, we suggest that these workflows may be a first step toward larger studies basing comparison of workflow systems on comparing implementations of the same task (e.g. statistics of a GFF file) in different workflow systems, as opposed to consideration of different general properties of workflow systems, as in [12,15].

Our work differs significantly from previous work on developing microarray data processing workflows. To our knowledge, our PCR primer design workflow is unique in that it is the first workflow that only requires basic genomic coordinates and organism to produce predicted primers with a graphical display of primer location in reference to other genomic features, such as single nucleotide polymorphisms (SNPs). For Nimblegen ChIP-chip GFF files, the Galaxy system is a web-based platform [11] offering a significant set of predesigned tools and workflows. In contrast to this web-based approach, our ChIP-chip workflows are entirely based on using local data sources and local programs. Unlike Galaxy, Kepler allows workflow components to be directly written in R, Java, Python, or an internal Kepler language, enabling rapid development and implementation of new functionality in microarray workflows. Overall, Kepler is designed to be a general purpose scientific workflow system supporting many models of computation [13], in contrast to Galaxy. In the Taverna system, there are a number of related workflows for Affymetrix microarray data processing [20]. These workflows are based on the Rserver/client model and rely heavily on remote data services. In contrast, we establish an Affymetrix microarray analysis workflow using local R scripting approaches (Rscript), without use of remote web/data services, and that features clear (and modifiable) display of parameter values on the workflow canvas.

## Implementation

### Availability of Kepler and the set of workflows

Kepler is freely available and open-source (http://www.kepler-project.org) and under active development. It will operate under Windows, Mac OSX, and also a large number of Unix-type operating systems (e.g. Linux). The community of developers is open to skilled personnel. Stable releases of the Kepler system and current development versions are available. Other programs included in the workflows are all open-source and freely available: Primer3 (http://primer3.sourceforge.net), ImageMagick (http://imagemagick.sourceforge.net).

All of the programming was performed in accordance with Kepler User Manual guidelines. All the workflows, required accessory programs, and detailed information on installation are available from http://chipanalysis.genomecenter.ucdavis.edu/Kepler1.0Package.zip and all programming code is included and accessible from the workflow files. Additional file 2: Table S 1 lists the operating systems used for development of each of our workflows. Many of these workflows will work under multiple operating systems, usually with no modification. Workflows have been developed to use the Kepler graphical interface for end user interaction.

### Kepler features relevant to microarray bioinformatics

Kepler is currently used in many areas of science requiring manipulation of large and/or complex datasets (e.g. [21]). General features of the Kepler system have been described [13] and an extensive user manual is available (http://www.kepler-project.org). A Kepler workflow for processing metagenomic data has previously been produced with some discussion of general Kepler features for bioinformatics [22]. Here, we highlight features of Kepler relevant to microarray bioinformatics pipeline design.

In brief, general features of the system include:

· Multiplatform. Kepler is available for Linux, Windows, and Mac OSX.

· Graphical. Workflows are presented in a simple graphical format that promotes rapid comprehension of inputs and logic. Elements of the workflow are added/removed, “grabbed”, moved, and connected in a graphical and intuitive manner.

· Built-in support for programming using various methodologies popular in bioinformatics, including R, Python (Jython), Java. BioConductor can be easily used via R.

· Presence of a simple internal language (like bash shell language [23]) for simple operations (internal Kepler language).

· Ability to integrate external program components (e.g. standalone programs).

· Ability to integrate Internet and website data sources and services.

· Workflows can consist of any combination of actor types (e.g. Java, website access, R in same workflow).

· Multiple levels of hierarchy. Modules (‘actors’) can be composed of other full pipelines (‘workflows’).

**Figure 1:**
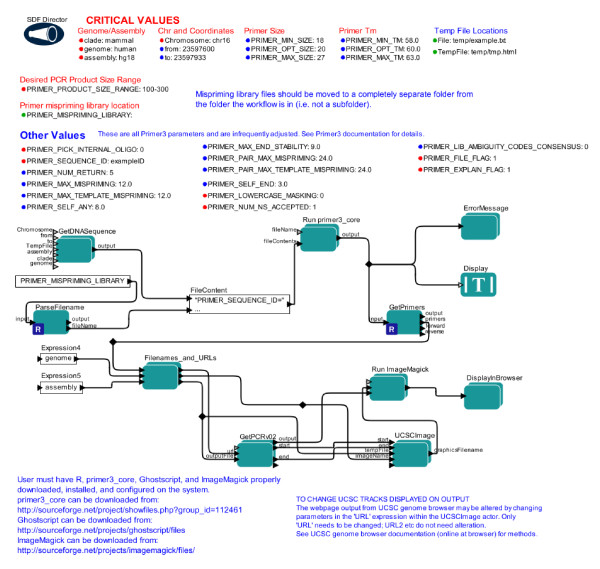
**Screenshot of a complex workflow for designing PCR primers.** By using locally installed software and online resources, this workflow can design primers for any genome available at the UCSC browser simply by requiring the user to provide genome assembly information and genomic coordinates. See RESULTS for details.

· Extensive annotation/note-taking capabilities. Multiple annotations (“text boxes”) can be placed anywhere on the Kepler canvas for clarity (e.g. Figure 1).

· Distribution of complete workflows as single small files, even if composed of different kinds of actors (e.g. Java and R actors). However, if external programs are invoked, these external programs need to be separately distributed.

Kepler uses one fundamental metaphor for information processing. Pipelines are viewed as a series of steps in which “work” is performed on the data (hence “workflow”). Each actor accepts data via input port(s), manipulates the data and then outputs the results via output port(s). Inputs and outputs can be single values, arrays of values, or complex objects. The “Director” specifies the overall nature of information flow through the system. We used the SDF director in all our workflows, but other directors may be appropriate in some cases (see [24] for detailed description). For clarity, we list the following relationships between traditional procedural programming and Kepler workflow programming:

· Kepler “workflows” are bioinformatics pipelines

· Kepler “parameters” correspond to global variables for traditional programming languages

· Kepler “actors” correspond to functions for traditional procedural programming

· Kepler “annotations” are similar to comments in a programming language

### End user system interaction

All workflows are designed to be used from the graphical interface instead of the command line. To execute a workflow, the end user will begin by starting the Kepler application and then loading the workflow into Kepler via the File menu. Workflow parameters are adjusted by clicking on the parameter as displayed on the workflow canvas, which will cause a box to be displayed in which the user may type the new parameter value (Figure 2). This new value will be immediately displayed on the workflow canvas. To guide the end user in system usage or appropriate parameter options, the bioinformaticist can place text boxes with instructions anywhere on the canvas and can control font, font size, and font color. This guidance is present in the majority of our workflows (see Additional file 1: Figures S1-S26). The user initiates operation of the workflow by pressing the “play” button.

**Figure 2:**
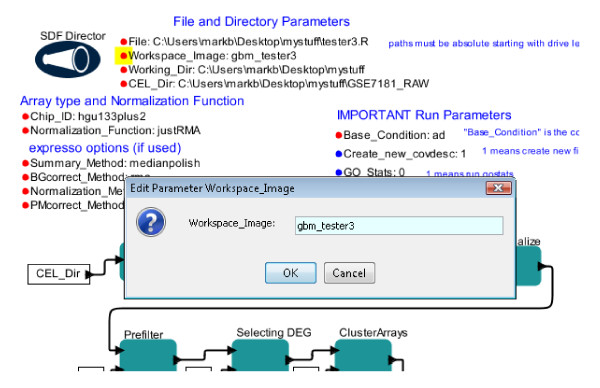
**Changing Parameter Values in Kepler.** This is a screenshot showing the changing of a parameter value in a workflow. Parameter names and values are displayed clearly on the Kepler canvas. Parameters may be easily moved into groups and font type, size, and color can be manipulated to provide visual cues as to importance or group relationships. Parameters are changed by simply clicking on the parameter. As shown here, clicking on the parameter indicates the parameter in question with a small yellow box and produces a text box in which the new parameter value may be entered. The displayed workflow is the gene expression workflow shown in Figure 5. See RESULTS for details.

### A model of bioinformatics usage in the laboratory

Workflow implementation is fundamentally based on an implicit (or explicit) model of use of workflows within the laboratory. Different environments (e.g. large genome centers vs. individual laboratories) may have different usage patterns and requirements. Our development approach is based on the following model of workflow usage. The experienced bioinformatician uses a library of actors and workflows to create custom workflows for specific applications. Some of these workflows may be reused often and rarely changed, but others will be frequently altered. This library of actors and workflows is provided by several sources, including myExperiment [25], the tools in this study, and custom produced actors created by the bioinformatician. The experienced bioinformatician will provide basic instruction in usage of the system, as happens commonly in laboratories with many applications. The end-user only changes the input data and a small number of other parameters.

## Results

### The set of 26 workflows

The full set of 26 workflows is presented in Table 1. Additional information is supplied in Additional file 2: (Table S 1). Full display of each of the workflows is presented in Additional file 1: Figures S1-S26. These workflows fit into four basic families. For Affymetrix gene expression microarray processing, there are two closely related workflows, differing by differential gene expression determination methodology. For PCR primer design, there is one workflow. There are four basic workflows implementing unix utilities for convenience. For GFF file processing aimed toward NimbleGen ChIP-chip data files, there are 19 workflows that are designed to span a wide range of laboratory needs for ChIP-chip analysis. These nineteen workflows can be subdivided into four further categories: Descriptive Statistics and File Information, File Modification, File Processing, and Binding Site Analysis (Table 1). “Descriptive Statistics and File Information” consists of nine workflows that compute and display basic statistics and information on GFF files. These workflows can be easily modified to pass this information as data instead of simply displaying it. The “File Modification” set consists of three workflows that perform basic modifications of GFF files for user convenience. Notably, the gffMakeTiny workflow greatly reduces the size of a GFF microarray data file in a lossless manner while maintaining the GFF format. The “File Processing” set consists of six workflows that are primarily concerned with sorting, smoothing, and quantile normalization of GFF files. Finally, the “Binding Site Detection” group consists of two workflows that implement the peak finding algorithm for ChIP-chip data (“Tamalpais”) that is described in [26]. One of these workflows (Run_Detection_with_annotation) also annotates the resulting list of peaks by using the R/BioConductor package ChIPpeakAnno [27]. The annotation consists of the identity of the nearest gene, distance to nearest gene, and peak relationship to gene transcription start site (see [27] for detailed description). Taken in toto, these GFF utilities provide a set of workflows/components that can be easily combined or altered. This is most clearly demonstrated by comparison of the gffQN_SM3_TINY workflow with the gffMakeTiny, gffSmooth, and QuantNorm workflows.

**Table 1:** Microarray Workflow Listing

**Workflow file name**	**Goal**
***GFF file workflows***	
	***Descriptive Statistics and File Information Group***
DisplayRegion.xml	Create a graphical display of the value field of a GFF file (like output provided by NimbleGen SignalMap)
GeneralHist.xml	Create a histogram of a given column of a text file. Useful for microarray GFF files.
gffFreqPoly_python.xml	Make several frequency polygons superimposed on one another for comparison.
gffFullDescription.xml	Display information about the GFF file specified.
gffQuickLook.xml	Displays first few lines of a GFF file.
gffStats_gffread_simple.xml	Calculate min, max, mean, median, num of lines, and various percentiles of a specified field. (Python version)
gffStats_Rbased_simple.xml	Calculate min, max, mean, median, num of lines, and various percentiles of a specified field. (R version)
ProbeSpacings.xml	Make a histogram of the probe spacings of a GFF file.
	***File Modification Group***
AddComments.xml	Add comments to the beginning of a GFF file.
gffMakeTinyl.xml	Greatly reduces the size of a GFF so that loading and processing is much faster. Reduces file size by replacing the second, third, and last fields of the file with placeholders. Assumes that these fields are the same in all lines.
gffModThirdField.xml	Modify the third field of a GFF file.
	***File Processing Group (Sorting, Smoothing, Normalization, Subtraction, Splitting)***
gffSmooth.xml	Median smooth (length 3) the 6th column of GFF files.
gffSort.xml	Sort a GFF file in chromosome + start point order (actually field 1 then field 4 order).
QuantNorm.xml	Quantile normalize the 6th field (ratio field) of a series of GFF files.
gffQN_SM3_TINY.xml	Quantile Normalize, Smooth, and Tiny-ize a set of GFF files. See gffMakeTiny.xml for explanation of Tiny-ize.
gffSubtract.xml	Subtract one GFF file from another GFF file (result based on subtraction of values in field 6).
gffSplit.xml	Split a GFF file containing the strings ‘tiled region’, ‘transcription_start_site’, and ‘primary_transcript’ into 3 separate files.
	***Binding Site Detection***
RunDetection.xml	Calculates runs of ratios (6th field) that are greater than or equal to the specified percentile of that column. Can be used for binding site detection for ChIP-chip as in [26].
RunDetection_with_annotation.xml	RunDetection workflow with added annotation of resulting binding sites (e.g. nearest gene) by using R/BioConductor ChIPpeakAnno package
***Affymetrix Analysis***	
AMDA.xml	Perform Affymetrix gene expression microarray analysis.
AMDA_limmafinal.xml	Variant of AMDA workflow using limma package [28] for differentially expressed gene determination.
***PCR Primer Design***	
PrimerDesign.xml	Pick sets of primers, given a chromosome range from user. Uses UCSC genome browser for outputs.
***General Utilities***	
Regex_R.xml	Simple example of find a substring within a string using regular expressions in R framework.
kepler_cut.xml	clone UNIX ‘cut’ command
kepler_paste.xml	clone UNIX ‘paste’ command
kepler_sort.xml	clone UNIX ‘sort’ command

These workflows are further described in Additional file 2: Table S 1. Each workflow is displayed in Additional file 1: Figures S1-S26.

**Figure 3:**
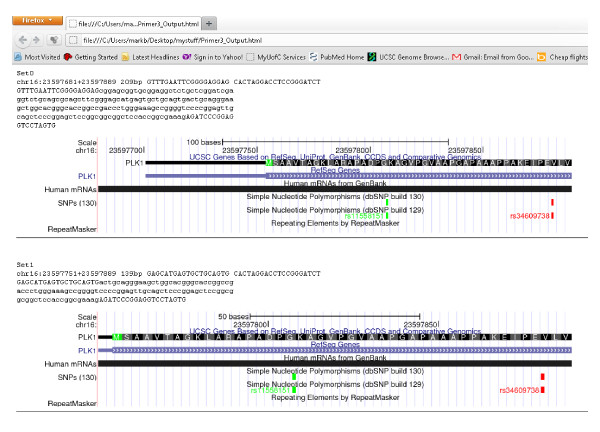
**One output of PCR primer design workflow.** Screenshot of partial graphical output of PCR primer design workflow as displayed in web browser. The output is truncated for representation clarity. This figure displays the first two primer sets generated for the region chr16:23,597,600-23,597,933 of the human genome (assembly hg18). The primers and PCR product are illustrated in text followed by graphical representation of PCR region derived from UCSC genome browser. Tracks displayed from browser may be changed by adjusting workflow.

The workflows associate data with workflow (a form of “data provenance”) using several strategies. For the GFF workflows, generally the output files follow a naming scheme indicating origin. In addition, some workflows present only displayed data, making this issue moot. For the Affymetrix gene expression microarray analysis workflows, both the R script file and RData file are stored using user-specified names in the directory with the analysis output files. The primer design workflow stores parameters from every run (primer3_output.txt, showing output of primer3, is created) and the output of the primer design workflow will directly indicate if primers are in the correct region (see Figure 3). Finally, in all cases, it is possible to save the workflow itself under a run-specific name.

In the following sections, we present a detailed review of three workflows (of the 26) across the range of our set (GFF processing, Affymetrix processing, and PCR primer design) to illustrate design and functionality.

### A simple Kepler workflow for ChIP-chip GFF file statistics

The GFF file format [29] is a widely used file format for genomics data. For NimbleGen ChIP-chip data, the value field of a GFF file stores the log_2_ enrichment value (log_2_ Chip-DNA channel/genomic DNA channel). In this workflow, we calculate basic statistics for this field. These basic statistics can indicate whether an experiment shows significant amounts of high enrichment.

**Figure 4:**
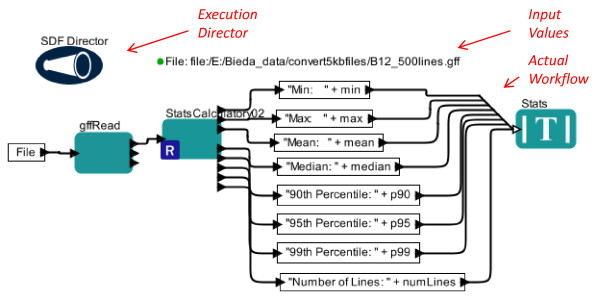
**Screenshot of a simple Kepler workflow for calculating statistics of the “ratio” column of microarray GFF files**. Python (Jython) and R implementation. Note that red arrows and italicized red text are not part of original screen view but are added to show basic parts of a Kepler workflow screen. All other text is part of screenshot. See RESULTS for details.

Figure 4 displays a simple Kepler workflow that calculates statistics on the value field (field 6) of a GFF file (specified by ‘File’). In this workflow, the ‘File’ parameter simply outputs its value to the input port of the ‘gffRead actor’, which is written in Python (Jython). This actor reads the file, then outputs only the 6th column to the R actor named ‘StatsCalculatorv02’. The ‘StatsCalculatorv02’ actor contains R code that calculates various statistics on the sixth column of the file and outputs the values. There is one output value per port (e.g. the top output port of ‘StatsCalculatorv02’ just outputs the min value). Then, these output ports send values to actors written in the internal Kepler language to append descriptive information. For example, the top output port of ‘StatsCalculatorv02’ sends its value (the min value) to the actor in the top box which simply prepends the string “Min:” to the value. Then, these output ports simply send values to the display actor, which displays results in a small window. Importantly, although this workflow uses multiple programming methodologies, it can be distributed as a single small file.

We chose to use a Python actor for gffRead because Python is much faster at reading large files than using read. Other methods of reading GFF files in R may allow large speed increases. An alternative version of this workflow  using  only  R  is  available  (gffStats_Rbased_simple) in our workflow set for comparison. Because Kepler allows the use of various programming approaches in the same workflow, this division of tasks across Python and R is natural to implement.

### A workflow for Affymetrix gene expression microarray analysis

**Figure 5:**
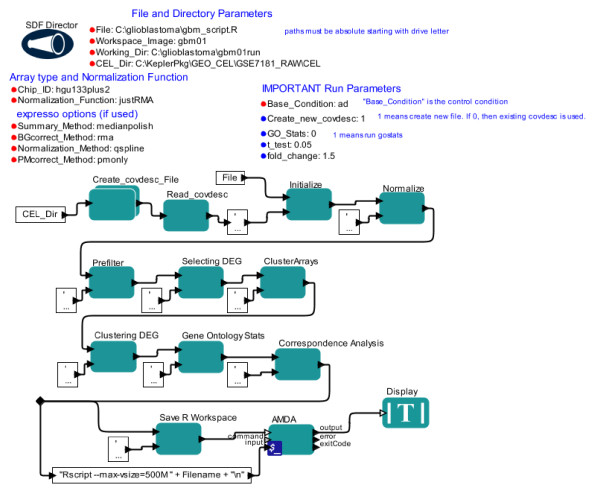
**A full Affymetrix gene expression microarray analysis workflow in Kepler.** This workflow uses well-established R/BioConductor modules following the steps recommended in a published pipeline [9]. Several resulting graphs and files are output. See RESULTS for details.

The workflow presented in Figure 4 represents a relatively simple task. In contrast, analysis of Affymetrix gene expression microarrays consists of several complex steps, with significant choices of options for each step. As a baseline for appropriate Affymetrix microarray analysis tasks, we chose to implement the basic steps of the existing R/BioConductor pipeline AMDA [9] within Kepler (Figure 5). AMDA essentially develops a custom set of functions as a convenient wrapper for a series of well-known and well-established R/BioConductor modules. AMDA produces a variety of outputs, including both graphs and lists of differentially expressed genes. To allow AMDA to accommodate larger numbers of microarrays, as is often used in current data analyses (e.g. [3]), we slightly modified the AMDA pipeline to eliminate some memory intensive steps and to use alternative modules that are less memory intensive. We implemented analysis for the widely employed experimental design of comparing test conditions to a control condition (e.g. microarrays that are ‘control’, other microarrays from ‘drug #1 treated’, other microarrays that are ‘drug #2 treated’); this design is referred to as ‘common reference’ or ‘common baseline’ in the AMDA description; see [9]. We developed two versions of this workflow that differ in determination of differentially expressed genes. The Figure 5 workflow displays a version that determines differentially expressed genes using t-tests as implemented in the R/BioConductor package simpleaffy [30], while the second version (AMDA_limmafinal; Additional file 1: Figure S 25) uses the package limma and allows determination based on adjusted p-value scores (see [28] for details).

**Figure 6:**
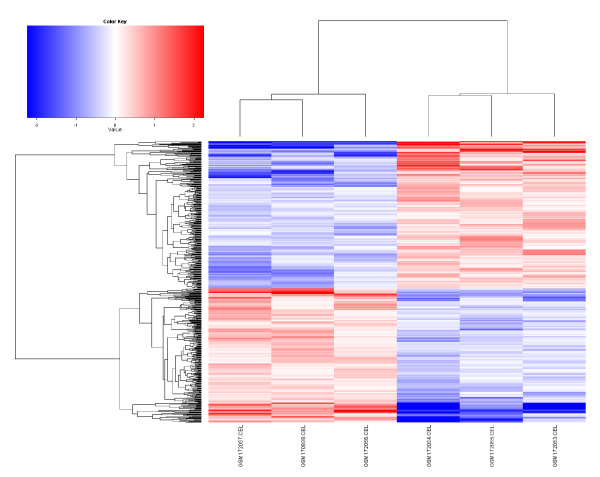
**One output graph from AMDA workflow applied to NCBI GEO dataset GSE7181.** One output of the Affymetrix gene expression workflow is displayed in Figure 5. This is a display of the file Data_norm_HeatMap.png, which is produced and stored by the workflow (see RESULTS). This is a heatmap representation of clustering of differentially expressed genes from six gene expression microarrays, each from a different line of brain tumor stem cells. The three adherent lines cluster together to the left; the three lines that grow as neurospheres cluster together to the right. Details of experimental methods and goals are found in [31] and [32] and heatmap generation methods details in [9].

The data output of this workflow consists of the workspace image, the produced R script file, and a series of files containing graphs and data. Graph and data files are stored in the location specified by the ‘Working_Dir’ parameter which is defined by the user in the ‘File and Directory Parameters’ section. Figure 6 displays the heatmap output (file Data_norm_HeatMap.png) of this workflow when the workflow was applied to a set of microarrays from NCBI GEO GSE7181 (see [31,32] for full description). Workflow output files are further described in the distribution package (Kepler1.0Package.zip; see Availability section of this report). The actual R script that is produced (see below) is stored at the location and with the file name specified as the ‘File’ parameter on the canvas, and the workspace image (.RData file) is stored in the ‘Working_Dir’ location starting with the name specified by the ‘Workspace_Image’ parameter; in Figure 6 the output file will be gbm_tester3.RData. Because these R script and RData files are created with each run of the workflow, the workflow is “autodocumenting” in that these files contain all the steps and parameters used to create the outputs.

The basic strategy is for each actor to add lines to an R script file that is then executed using the command line R program (Rscript; actor ‘AMDA’). The steps are as follows. First, it is necessary to assign each microarray to a group (e.g. ‘control’ microarrays vs ‘drug’ microarrays). The actor ‘Create_covdesc_File’ is a composite actor: it is composed of a workflow that includes a Java actor using the Swing toolkit to allow a simple window interface for the user to enter information for each microarray, creating a small file often referred to as a ‘covariant description’ or ‘covdesc’ file in the R module literature. Second, after initialization and loading of microarrays (actor ‘Initialize’), the actor ‘Normalize’ performs normalization of microarrays. Normalization can be memory intensive [33]. The relatively low memory usage and fast justRMA function is a standard choice for both small and large microarray datasets. For purposes of reproducing older published analyses, the R/BioConductor function expresso can be chosen (‘Normalization_Function’ parameter) and subparameters can be set (‘expresso parameters (if used)’ group). Third, the microarray set is “prefiltered” to eliminate probesets that are called as ‘absent’ on all microarrays, as these probes are considered uninformative (actor ‘Prefilter’). Fourth, differentially expressed genes (DEGs) are found using simple *t*-test and fold-change criteria, as are commonly used (see Chapter 6 of [34]). These values are established in ‘IMPORTANT Run Parameters’ in actor ‘Selecting DEG’. Use of fold-change, in particular, may be valuable when only small numbers of microarrays are available (e.g. n = 2 in each condition) and are intended to be analyzed to provide candidate target genes. Given the range of DEG selection approaches commonly employed, the DEG selection approach should be carefully examined (see [34] for a partial listing and rationale for different DEG selection approaches). The DEG selection approach can be easily altered by the experienced microarray bioinformatician by altering the ‘Selecting DEG’ actor to use different R/Bioconductor packages. For use of adjusted p-values (as implemented and suggested in the R/Bioconductor limma package [28] for gene expression microarray analysis), we have created a second gene expression workflow (AMDA_limmafinal; Additional file 1: Figure S 25). This second workflow has parameters on the canvas for choice of adjustment method of p-values and, by comparison with the Figure 5 workflow, illustrates directly how the DEG selection method can be changed. Fifth, independently of DEG selection, the microarrays are clustered using hierarchical clustering and Pearson correlation as a distance measure, an important step for determining potential outlier microarrays and overall relationships (actor ‘ClusterArrays’). Sixth, DEGs are clustered to determine groups of genes with similar expression profiles across conditions (e.g. genes that are highly expressed in control and downregulated with drug application; actor ‘Clustering DEG’). Seventh, gene ontology (GO) statistical significance is computed for the genes in each DEG group (actor ‘Gene Ontology Stats’). Eighth, correspondence analysis, a data reduction approach for high-dimensional data, is performed (see AMDA documentation; actor ‘Correspondence Analysis’). The workspace is saved by actor ‘Save R Workspace’. Finally, the last actor (‘AMDA’) invokes command line R (Rscript.exe) to run the full script.

Errors are indicated in several ways in this workflow. Simple errors, such as having an incorrect name for the directory containing the CEL files, produces a large red box around the offending actor. Furthermore, the workflow at its conclusion displays a window that resembles the CMD program window in Windows. Rscript is run from this window, and just as in the case of using a CMD window, will produce the text output of the script, which can indicate where execution ceased. This raises the possibility of employing the classic error finding methodology of strategically placed print statements to determine the exact point of occurrence of a problem and to monitor variable values.

There are several notable features of this workflow. First, this workflow demonstrates some advantages of transforming an R/BioConductor pipeline into a Kepler workflow. Most importantly, the overall steps in the workflow are clearly illustrated without being buried in a long text file, as in a R/BioConductor script. Unlike a script representation, this graphical representation clearly separates functions from parameters. The parameters are grouped into different sets and notes have been added to indicate functions. Second, this workflow uses a composite actor - an actor that itself is a complete workflow. This allows simple clear packaging of complex tasks. Third, the workflow is “autodocumenting” because the generated R script is automatically saved, as is the R workspace. Hence, it is straightforward for experienced R users to investigate the actual exact steps used in the analysis and to retrieve all the parameters used for a given analysis outside of the Kepler environment. Also, optionally saving the Kepler workflow file (which is a small file) separately for each analysis will allow simple, rapid inspection of analysis parameters; the parameters are presented in a natural set of categories, allowing quick and intuitive review. Fourth, this workflow demonstrates the strength of Kepler in integrating diverse programming methodologies: Java + Swing is used for the input of the covdesc file, then the workflow creates the R script using the Kepler internal language, and finally the R application Rscript is invoked as a local application. Fifth, we found that an alternative implementation of these steps led to several tradeoffs. We initially implemented each step as a separate R actor. Because the R actor starts and stops R each time the actor runs, we found this solution to be much slower than using the “create full script then execute script” approach shown in the figure. Finally, the graphical display of the workflow highlights functional modules, allowing rapid discernment of the appropriate actor to alter for modified functionality. For example, if a different gene ontology statistics package were required, it is clear that the step labeled “Gene Ontology Stats” should be changed. Similarly, various collections of well-developed modules for bioinformatics (e.g. BioPerl, BioPython) could be quickly implemented using this general workflow design paradigm. Hence, existing, well-tested and understood collections of modules for bioinformatics can be easily moved into the Kepler environment. Furthermore, moving existing R/BioConductor pipelines into the Kepler environment allows clear display of functional units and clear separation of parameters from functions.

### Automated design of PCR primers for microarray validation experiments

PCR primer design is a very common task in molecular biology and the usual required step for validation of microarray results (e.g. [35]). Although there are valuable databases of PCR primers for some common tasks (e.g. gene expression level measurement via qRTPCR; [36]), these resources only address a portion of typical PCR primer design needs. For example, primer design for promoter and enhancer regions must often be performed *de novo* (e.g. [26]). A typical manual protocol for design of primers to a desired region in the human genome is:

(1) Access UCSC browser and retrieve the DNA sequence for the desired region.

(2) Copy and paste this sequence into the Primer3 website. Adjust the primer3 primer-picking parameters to use required values.

(3) Copy the set of resulting suggested primer pairs.

(4) For each primer pair, access the UCSC browser and perform in-silico PCR function to determine the exact coordinates of the resulting desired PCR product, that the primers do not overlap significant SNPs, that they do not overlap repeats, and that they do not map to other locations in the genome. Repeat for each primer pair.

(5) Repeat steps 1-4 for each different region requiring PCR primers.

For microarray experiments, there may be a relatively large number of regions to validate via PCR experiments. Hence, automation of this procedure is advantageous.

Figure 1 displays the final workflow. Our workflow involved website access (UCSC genome browser) and invocation of locally installed programs (Primer3, and, for final image processing, ImageMagick). As with Figure 5, we note Kepler’s ability to group parameters and provide informative notes and headings. The workflow follows the steps listed above:

(1) Retrieve sequence from UCSC browser (actor ‘GetDNASequence’). Notably, this actor can be used in any workflow to grab any sequence from the UCSC browser.

(2) Produce predicted primer sets by running locally installed Primer3 using sequence from ‘GetDNASequence’ using appropriate Primer3 parameters (listed on canvas). A single appropriately formatted input file is created by actors ‘ParseFilename’ and ‘MakeInputFile’ and then passed to Primer3 (‘Run primer3_core’).

(3) Submit each primer set to the UCSC genome browser to produce a graphical output showing locations and SNPs from the browser. ‘GetPrimers’ parses the primer3 output file to derive the primer sets. Because the UCSC browser uses GET calls for *in-silico* PCR, the workflow constructs HTTP calls and executes them to get the resulting images (actors between ‘GetPrimers’ and ‘UCSCImage’). The UCSC images (in encapsulated postscript format) are then passed to ImageMagick for reformatting as PNG files, which are then displayed in a webpage format (‘DisplayInBrowser’).

This workflow produces several output files. One graphical output of this workflow is displayed in Figure 3. There are several notable features of this workflow. First, the graphical design and clear naming of actor inputs and outputs allows conceptual clarity promoting the reuse of actors and sections of this workflow. For example, only casual inspection of the workflow is required to discern that ‘GetDNASequence’ can be reused to grab DNA sequences from the UCSC browser for other bioinformatics pipelines (e.g. grabbing promoter sequences for motif finding). Similarly, it is clear that the workflow segment after ‘GetPrimers’ could be used in other workflows for performing *in-silico* PCR using the UCSC Genome Browser and that the ‘UCSCimage’ actor can be used to grab “screenshots” from the UCSC Browser. Because the UCSC browser contains data from a wide variety of sources [37], this actor would be useful for targeted, automatic grabbing of screenshots of regions of the genome for many uses.

## Discussion

The growth in size, types and subtypes, and overall number of large scale ‘omics datasets has led to a large increase in the diversity, complexity, and rate of change of bioinformatics analyses. In turn, these complex and evolving needs have led to an increasing emphasis on development of analysis pipelines. Here, we developed a series of workflows that addressed microarray data set analysis. Microarray analysis pipelines are complex, subject to alteration with the development of new analytical tools, and also growing in complexity due to the development of new types of resources for output analysis, such as gene pathway systems [38], and new demands for integration with other sources of data (e.g. gene expression data with metabolomic data - [39]). We found that the Kepler system has functional and appropriate capabilities for microarray data analysis pipeline creation. The Kepler workflow model, and in particular its graphical implementation, provides significant advantages for pipeline creation by promoting conceptual and visual clarity. Finally, Kepler workflow programming allows a variety of overall design approaches; we create microarray data analysis workflows using several types of programming approaches.

Microarray data analysis will become more complex in the future. First, there are increasing current needs for microarray analysis involving integration of data from different gene expression platforms (e.g. integrating data from Illumina and Affymetrix gene expression systems) and different designs (e.g. exon microarrays; [3]). Analyses involving different platforms will require much more extensive analysis pipelines. Also, there are increasing needs for comparison of gene expression data with ChIP-chip or ChIP-seq (chromatin immunoprecipitation followed by sequencing) and comparative genomic hybridization (CGH; to determine copy number variation in genomic segments) datasets, which again will require more extensive pipelines [3]. As analyses become increasingly complex, it will become critical to use workflow systems to allow modifiable, extensible, and easily comprehensible analysis pipelines. We suggest that the workflows produced in this report represent a critical set of components for these future, more extensive pipelines.

Kepler features such as (1) actors with clear inputs and outputs and (2) the graphical nature of workflow development and representation offered significant advantages to us as compared to traditional shell scripting. For example, simple visual inspection quickly reveals that the gene expression workflow (Figure 5) includes a “correspondence analysis”, which is an optional step in gene expression workflows. The approach for eliminating this step is also clear: simple removal of the actor. Similarly, if the method for determining differentially expressed genes (DEGs) should be changed, the workflow visually indicates the correct actor to alter - the ‘DEG selection’ actor must be modified. Most importantly, we found that Kepler offered special advantages for bioinformatics pipelines involving a large number of potential parameters: even a brief glance at the gene expression workflow (Figure 5) or the primer design workflow (Figure 1) shows which parameters are usually changed and which parameters are usually kept constant. This is enabled by the nature of the Kepler canvas using positioning, text font, text size, and text color to organize parameters into clear visual groups.

Kepler enables a large number of potential software design strategies. This seems to be an advantage in that there is not necessarily a single best design approach for bioinformatics pipelines. For example, an actor could be implemented as a large block of Python code or, alternatively, as a composite actor embodying a workflow. Both implementations of this actor could look identical from a usage standpoint. Overall, the Kepler model favors actors passing data to each other. However, in some cases (e.g. Figure 5), we may wish to have the actors simply compose a script to be run externally. Kepler can easily support these different software design paradigms. This means that existing shell scripts and pipelines can often be easily moved into the Kepler system. Hence, a set of useful Kepler workflows and reusable actors can be quickly produced.

Runtime workflow errors are indicated in Kepler with a red box around the actor that encounters the error. This has the advantage of the indicating clearly which stage of the workflow needs to be investigated. However, the actual error output that is passed through Kepler is phrased in programming terms, which will be difficult to interpret for the end user. This problem is shared with many bioinformatics pipelines that rely on simple shell scripting and errors in R/Bioconductor can also be difficult to understand. This problem can be addressed by having the experienced bioinformatician add error-catching tests to the pipelines to produce informative error outputs.

Kepler offers advantages and disadvantages compared to other bioinformatics pipeline development approaches. In the much used “shell-scripting” approach, a series of external programs are invoked. Shell scripting has the advantage of being a well-known and easily accessible approach that can produce very rapid development, in particular for small tasks [23]. However, shell scripts for complex tasks can require careful study to understand. Furthermore, the mixture of programs and parameters at the point of invocation makes changing parameters confusing. Finally, packaging of shell scripts for distribution involves finding each subprogram and dependency. In contrast, Kepler workflows are often single files because the R, Python, Java etc. code is included in the workflow file (but external programs must be included as separate files). Similarly, developing pipelines within a single programming framework (e.g. BioPerl or R/BioConductor) is attractive for the knowledgeable programmer, but can be very difficult for the outside user to follow, extend, or modify. A second major approach is the use of web-based pipelines. These pipelines often indicate the analysis steps and parameters clearly (e.g. [10]), feature user interfaces (webpages) that are already known to the user, and can feature attractive, informative outputs that document the parameters that were used. However, these pipelines are usually inaccessible for modification and extension. Also, upload of large datasets to the servers can be slow and problematic.

A third option is the use of other workflow systems (e.g. Taverna: [14]; jORCA: [17]). Workflow systems generally share many properties (for review, see [12,15]). Hence, selection of a system may rely on specific features of the system. Taverna has been used for many bioinformatics pipelines with a strong emphasis on use of web services [25]. However, microarray analysis currently features relatively large primary data files and the file sizes are increasing in many cases (e.g. NimbleGen ChIP-chip experiments now are available in 2.1 million probes/array formats). Moving large files from internet site to site, as is necessary for Web services, is usually slow and can be error-prone, producing issues with speed of execution and effectiveness of using web-service oriented frameworks in this exact application area. Hence, we strongly prefer microarray workflows featuring local resources (data and programs). In addition, the published Taverna workflow system for Affymetrix microarrays [20] uses a more complex Rclient/server model rather than the simple local invocation of R in our workflows. Our use of R is the basic use introduced in R courses and books (e.g. [34]). Therefore, we believe that our approach is conceptually simpler, has a simpler implementation, and is more familiar to the majority of bioinformaticians, making this workflow easier to comprehend. Also, Kepler displays the parameters and parameter values simply and clearly on the actual canvas with the workflow, and changing these values is very simple (click on value on screen, change value, new value is shown on screen). We strongly prefer this clear display of parameters, which is not present in Taverna workflows. Finally, Kepler has a well-developed provenance module that has great promise for bioinformatics workflows.

Galaxy [11] is an increasingly popular platform for bioinformatics analyses. At this time, there are no gene expression microarray analysis capabilities in Galaxy. To bring new tools into Galaxy, a description of the tool is required to be produced. In contrast, external tools (e.g. local standalone programs) can be simply and directly invoked within Kepler actors without any need for descriptions of allowable inputs, outputs, or other formats. Furthermore, the current Galaxy canvas does not display parameters with values, unlike the Kepler canvas. Therefore, Kepler offers significant advantages in that parameter values are directly displayed and easily changed and also it is much quicker to integrate existing tools into Kepler. Workflows in this report often invoke external programs and provide many examples of this approach. Finally, Galaxy is based on a web model instead of being a simple standalone program like Kepler. Using Galaxy from a remote location can produce significant inconvenience of uploading data to a server, and a local installation of Galaxy will still use a web interface, a more complicated arrangement than the simple, standalone program that is Kepler.

Kepler has some disadvantages. Like other scientific workflow systems, using Kepler requires a time investment in learning the Kepler environment and the Kepler approach to actor creation. We expect that our production of a series of working bioinformatics pipelines will enable this process. Second, we found that the theoretically best approach for Kepler development - passing data as structures (e.g. arrays or objects) - led to some problems with execution time (e.g. Affymetrix gene expression workflow). We were able to develop a straightforward solution to this issue (creating a script then executing). Finally, there are far fewer prepackaged bioinformatics resources in Kepler as compared to Taverna, but the bioKepler project [40] should change this situation.

Making Kepler workflows more widely accessible, e.g., through popular workflow repositories [25], will allow pipeline developers to share workflows more easily, learn from each other, and thus make pipeline development increasingly straightforward

## Conclusions

The set of 26 workflows provides a solid foundation for ChIP-chip and Affymetrix gene expression microarray processing and subsequent validation using PCR experiments. We present the first workflow that designs PCR primers using any available genome/genome assembly in the UCSC genome browser and provides a graphical output indicating positions of known SNPs and repetitive elements in the PCR product. Transforming R/BioConductor pipelines into Kepler is relatively straightforward. Kepler offers many advantages over traditional scripting approaches for microarray data processing pipelines, with increasing advantages as these pipelines grow more complex.

## Availability and requirements

· Project name: Kepler Microarray Workflows

· Project home page:http://chipanalysis.genomecenter.ucdavis.edu/Kepler1.0Package.zipThis zip package contains all workflows and instructions for installation.

· Operating system: Windows, Linux, Mac OSX. Some workflows will only function under Windows; see zip package and supplementary table for details.

· Other requirements: Java 5, R/BioConductor, Kepler. See instructions in zip package for installation.

· License: MIT open-source

· Kepler is available at: http://www.kepler-project.org

## Competing interests

The authors declare that they have no competing interests.

## Authors’ contributions

TM, BL, and MB developed overall concept for project. MB developed list of workflows and programming approach, supervised and advised on all phases of project, tested and wrote some workflows, and wrote manuscript. TM and BL edited manuscript. TS created workflows and wrote underlying included programs. All authors have read and approved the final manuscript.

## Supplementary Material

Additional file 1 **Stropp et al. Additional file 1: Figures.pdf.** This file contains Additional file 1: Figures S1-S26 showing screenshots of all workflows listed in Tables 1 and S 1. Each figure includes some additional information on goals and usage. Click here for file

Additional file 2 **Stropp et al. Additional file 2: Table.pdf.** This file contains Additional file 2: Table S1, which is an expanded version of Table 1 including details of implementation and operating systems among other information. Full information on installation and running workflows is listed in the “Availability” section above. Click here for file
