# Supplementary material for: Workflows for microarray data processing in the Kepler environment
Source: BMC Bioinformatics. 2012 May 17;13:102. doi: 10.1186/1471-2105-13-102 (PMC3431220; doi:10.1186/1471-2105-13-102)
Supplement: Additional file 1 — Stropp et al. Additional file 1: Figures.pdf. This file contains Additional file 1: Figures S1-S26 showing screenshots of all workflows listed in Tables 1 and S 1. Each figure includes some additional information on goals and usage. [file 1471-2105-13-102-S1.pdf]

Stropp *et al.*
Workflows
Supplementary Figures

AddComments.xml

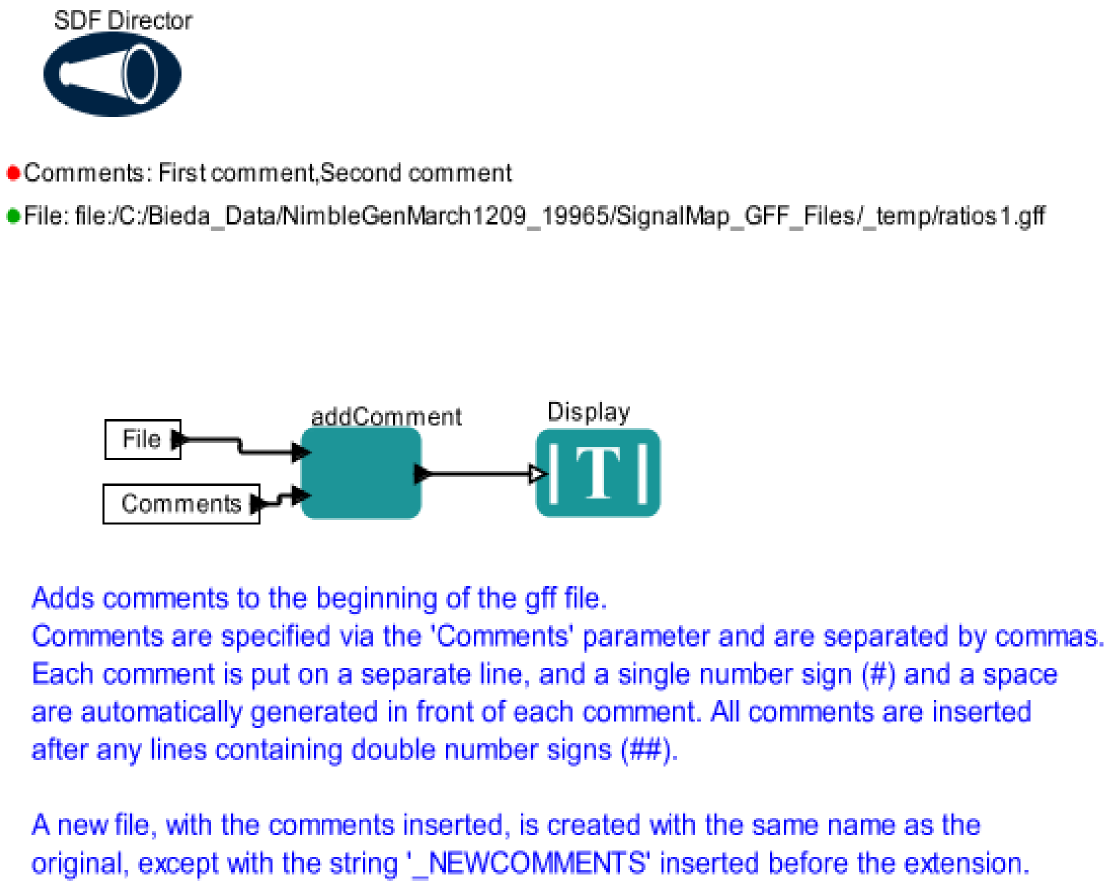

Fig. S1. AddComments.xml workflow.

AMDA.xml

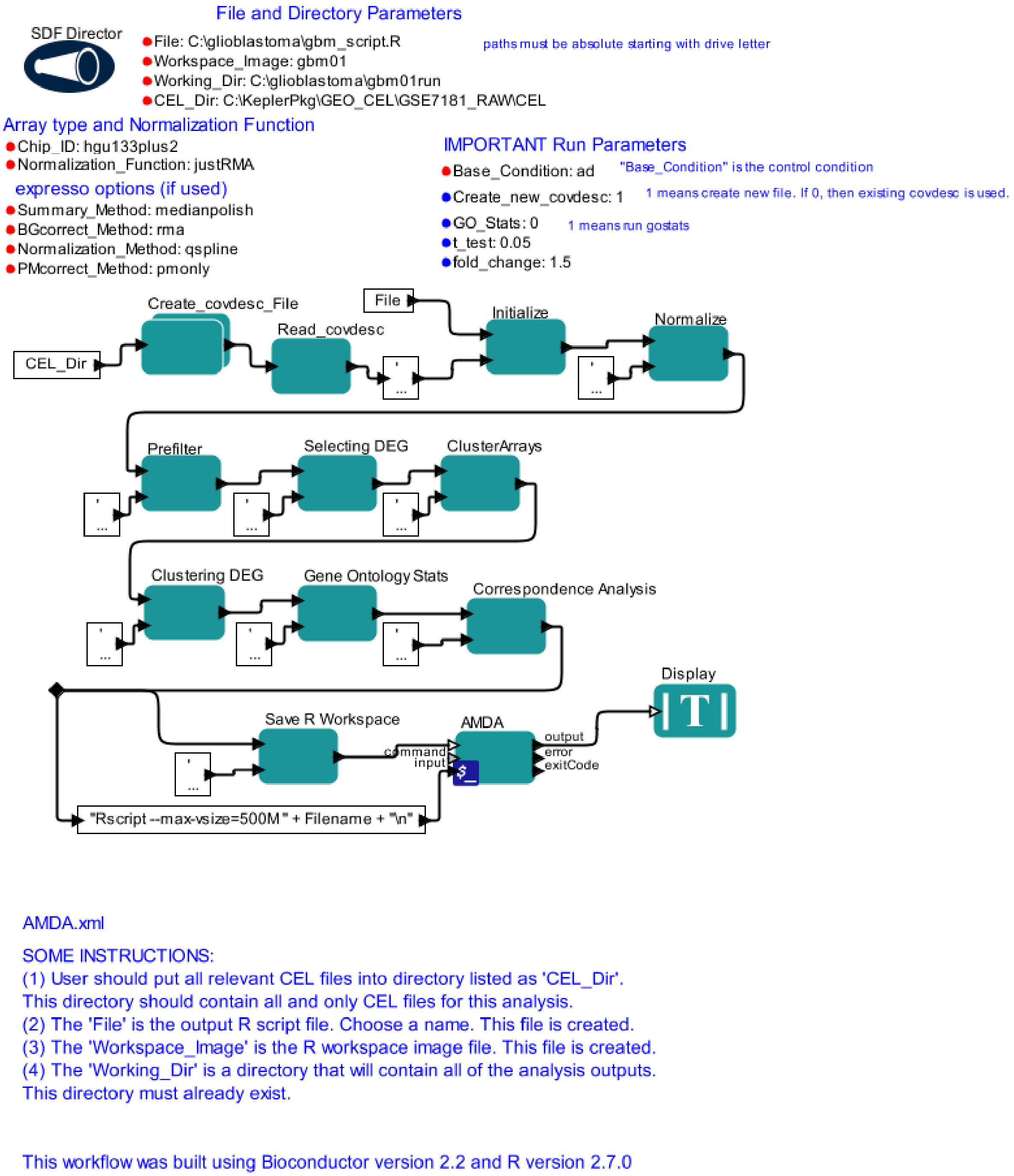

GOAL: Affymetrix gene expression pipeline creation (Fig 3 of Stropp et al).
Dependencies: R and BioConductor must be installed.

Fig. S2. AMDA.xml workflow. NOTE: there is a limma-based version available (Figure S25).

DisplayRegion.xml

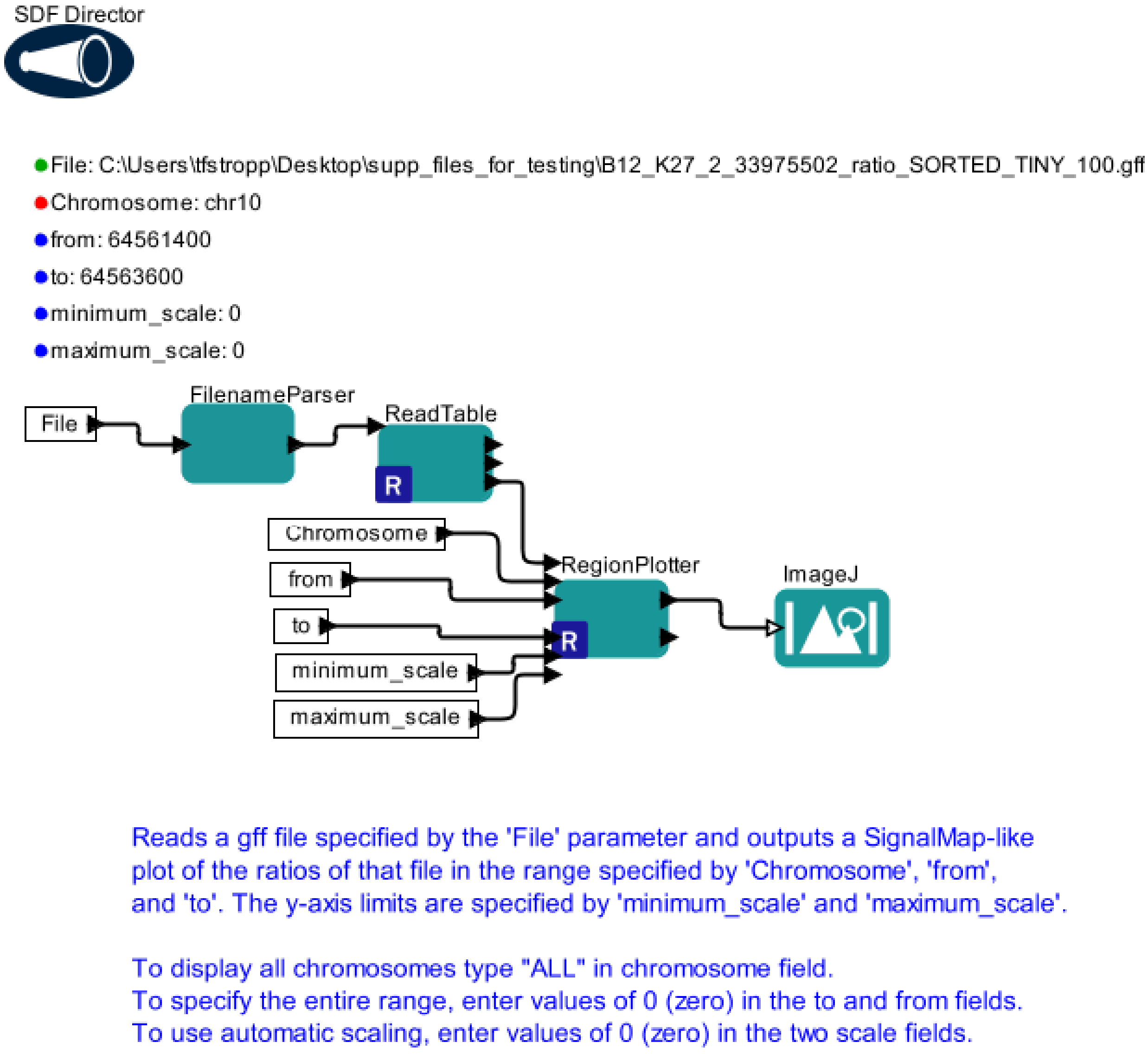

Fig. S3. DisplayRegion.xml workflow.

GeneralHist.xml

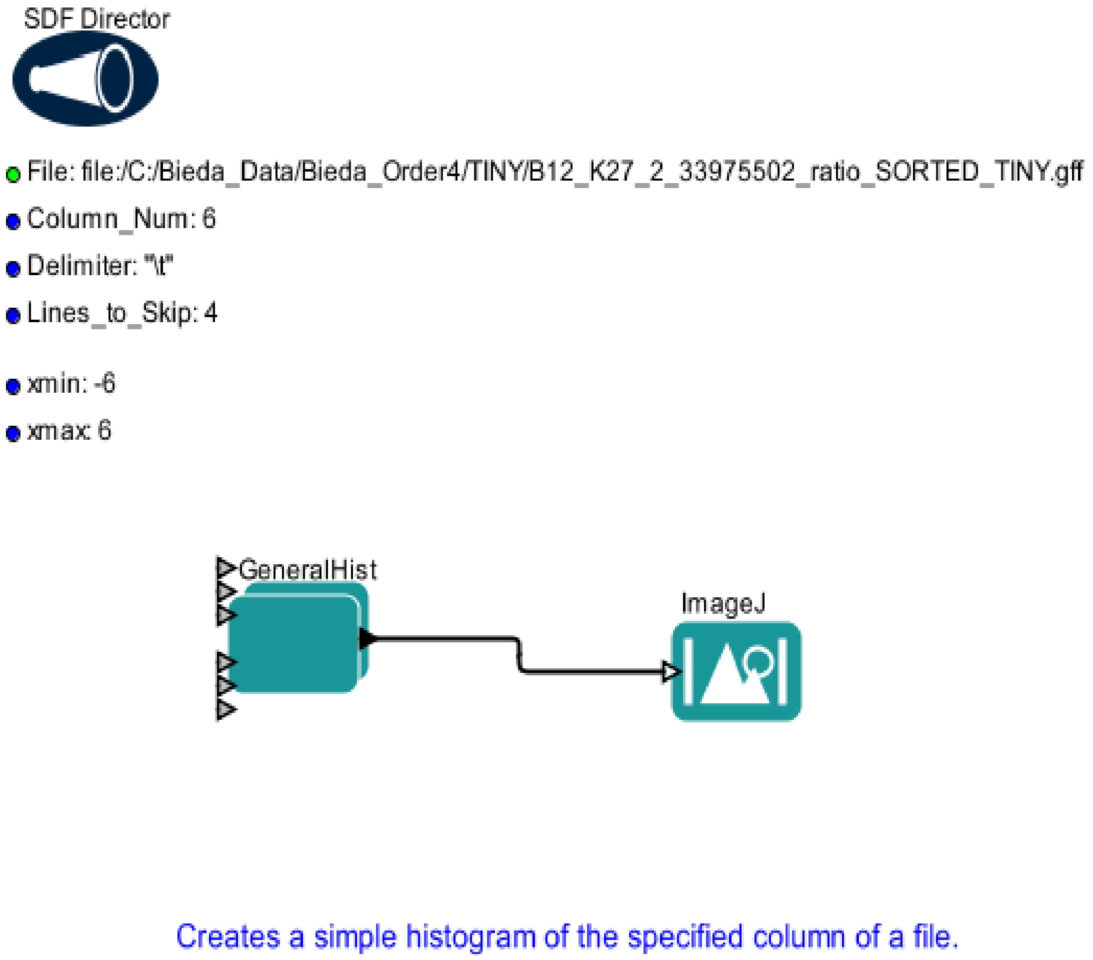

Fig. S4. GeneralHist.xml workflow.

gffFreqPoly_python.xml

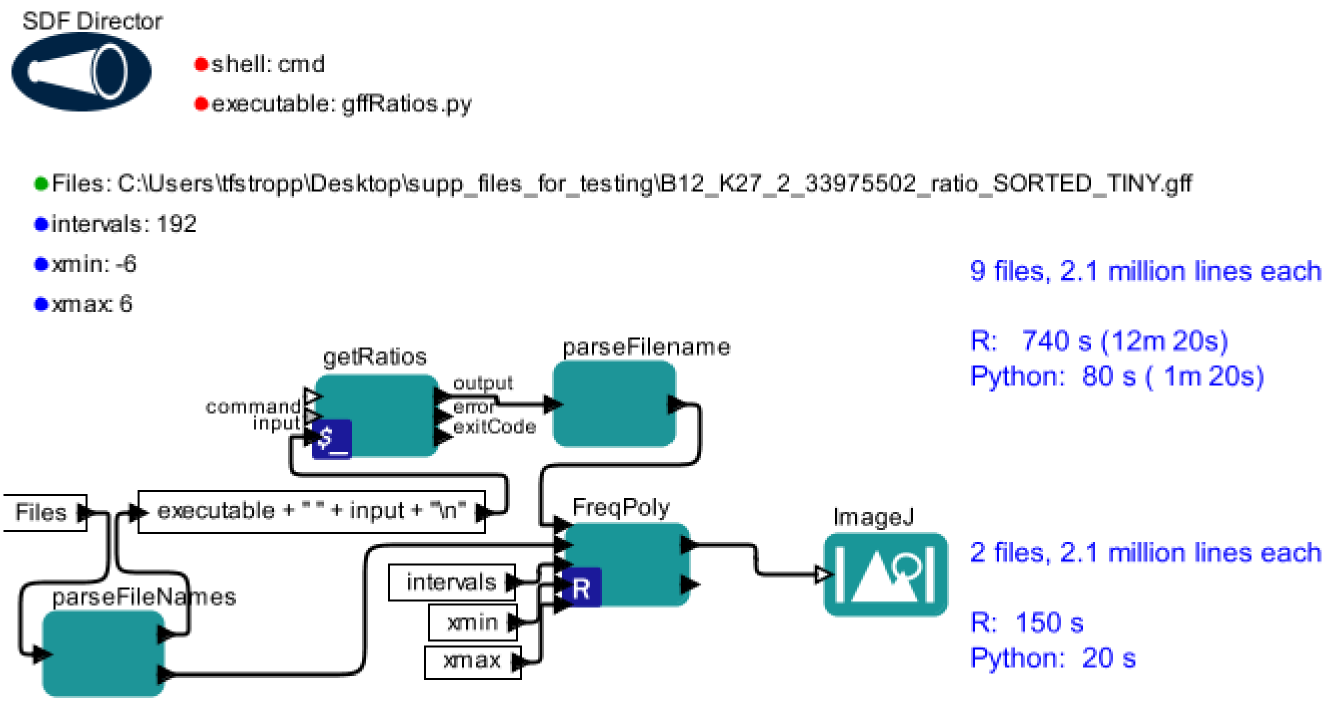

Reads a number of gff files and creates a set of frequency polygons superimposed on top of each other. The number of files it can read is limited only by the number of different symbols that can be used as points (currently 19), defined by an array called 'pnts' within the FreqPoly R actor. The workflow uses an external python program (gffRatios.py) because it runs considerably faster than the integrated python actors. The frequency polygon is displayed on screen and saved in the users ".kepler" folder.

File are specified via the 'Files' parameter, separated by semicolons (;). 'xmin' and 'xmax' are obviously the x value limits. They will automatically be modified if there is a value found to be outside the range specified. And 'intervals' is the number of intervals between xmin and xmax.

'shell' is a string specifying which type of shell interpreter is being used: generally "sh" if using Linux and "cmd" if using Windows.

Fig. S5. gffFreqPoly_python.xml workflow.

gffFullDescription.xml

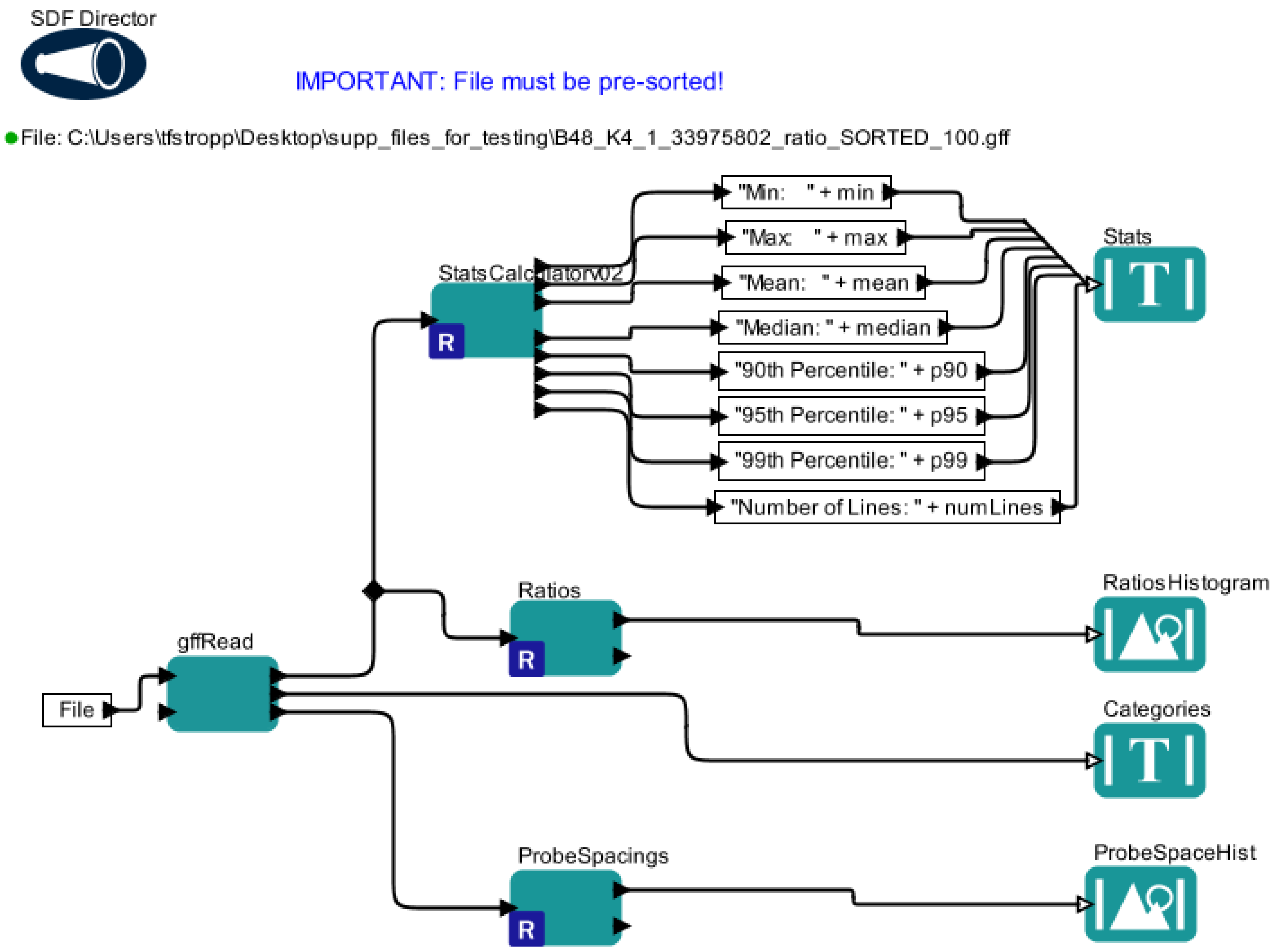

GOAL: Give “full” descriptive information on a GFF file.

Fig. S6. gffFullDescription.xml workflow.

gffMakeTiny.xml

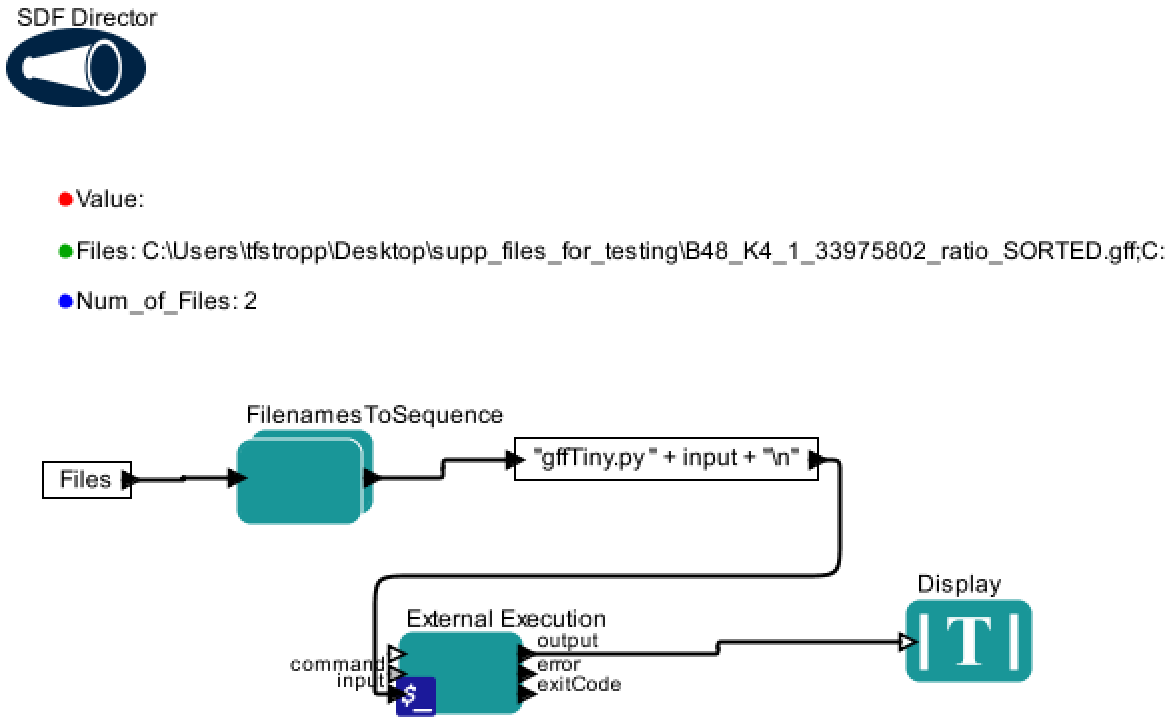

Fig. S7. gffMakeTiny.xml workflow.

gffModThirdField.xml

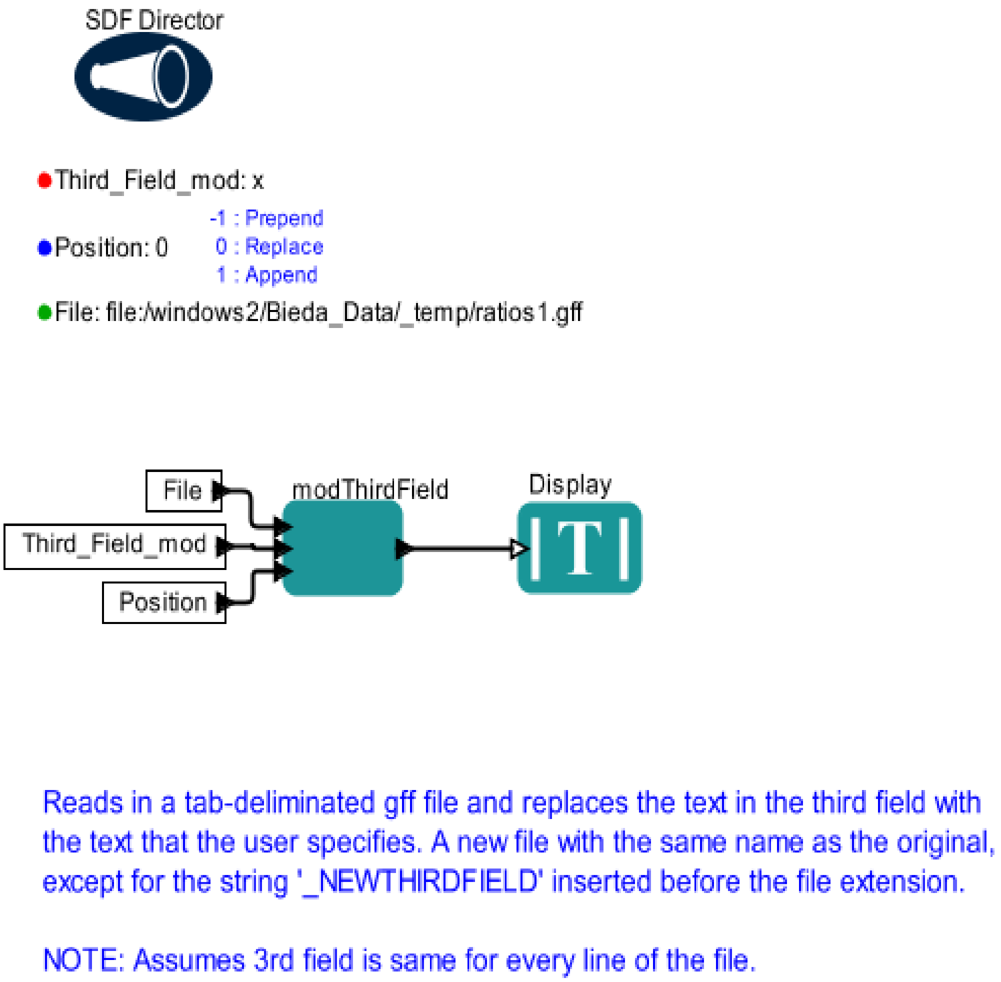

Fig. S8. gffModThirdField.xml workflow.

gffQN_SM3_TINY.xml

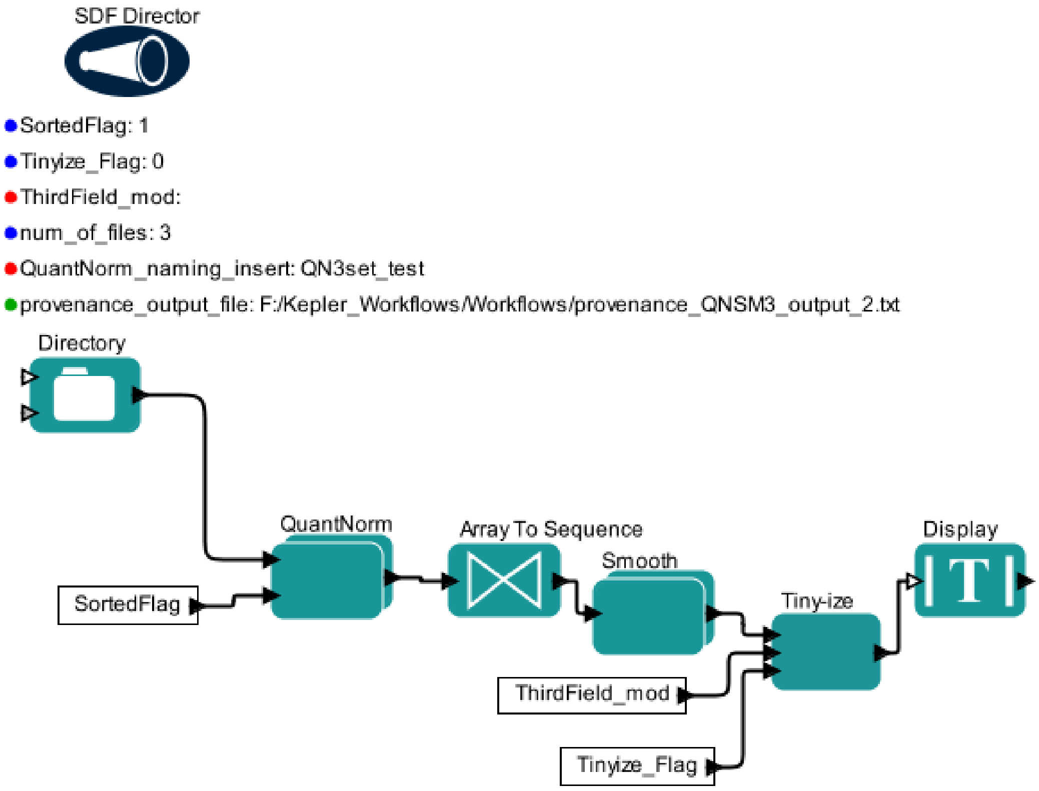

This workflow requires that the data be sorted beforehand if the files being manipulated are too big. My tests show that a 400,000 line file can be sorted, but a 2 million line file is too big.
The workflow will automatically create a new file after each step -- sorting (inserts '_SORTED' before file extension), quantile normalizing (inserts QuantNorm_naming_insert), smoothing (inserts '_SM3'), and tiny-izing (inserts '_TINY'). So up to 4 new files are created for each file being manipulated. It NEVER overwrites the original files.

Set SortedFlag to 1 if the files are already sorted, otherwise 0.
Set Tinyize_Flag to 1 if the files need to be tiny-ized, otherwise 0.
ThirdField_mod is what the new third field of the tiny-ized files will be. If left blank, the filename will be used.
num_of_files is the number of files that are being manipulated. If the number of files being passed from the Directory actor is not equal to num_of_files, an error will be thrown and the workflow will be halted.
Directory is the directory containing the files to be manipulated. There should be no other files in this directory.

Fig. S9. gffQN_SM3_TINY.xml workflow.

gffQuickLook.xml

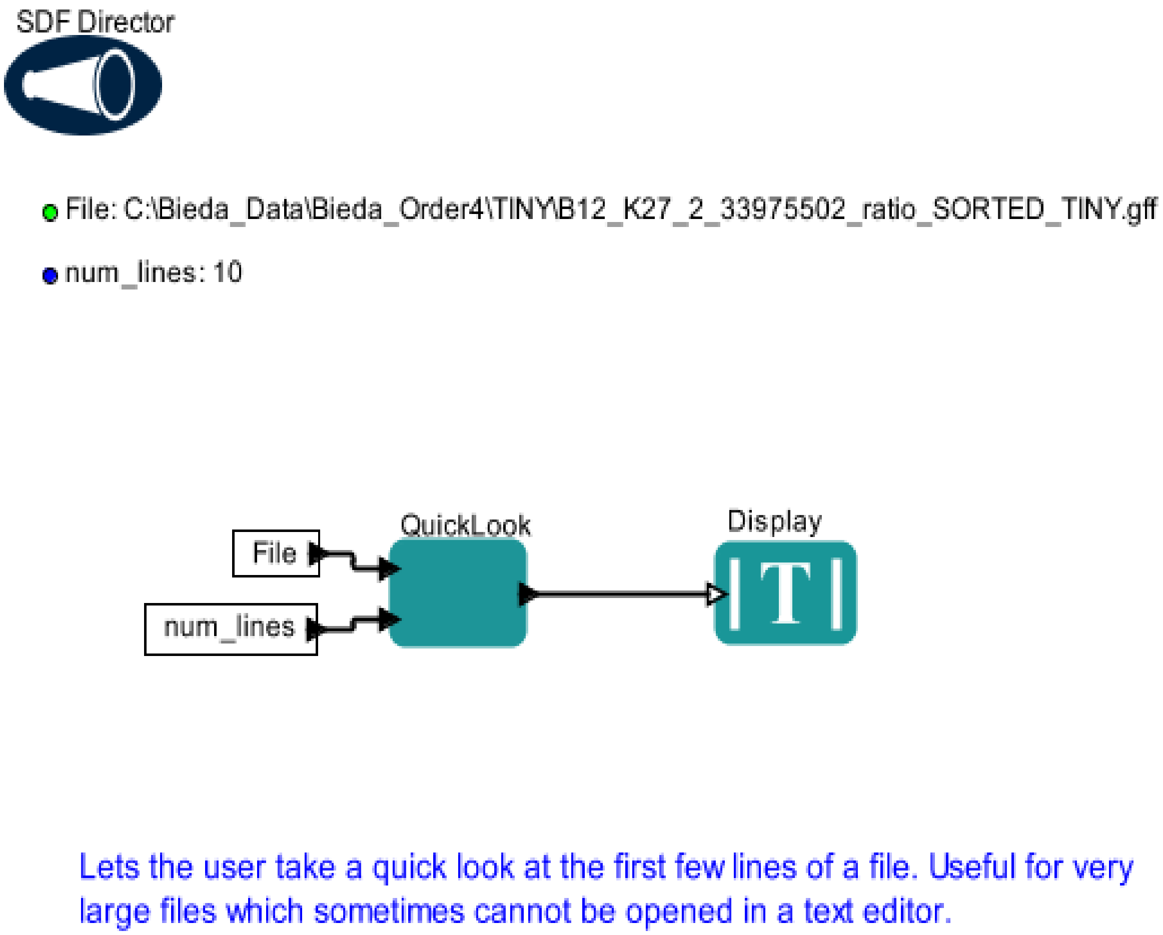

Fig. S10. gffQuickLook.xml workflow.

gffSmooth.xml

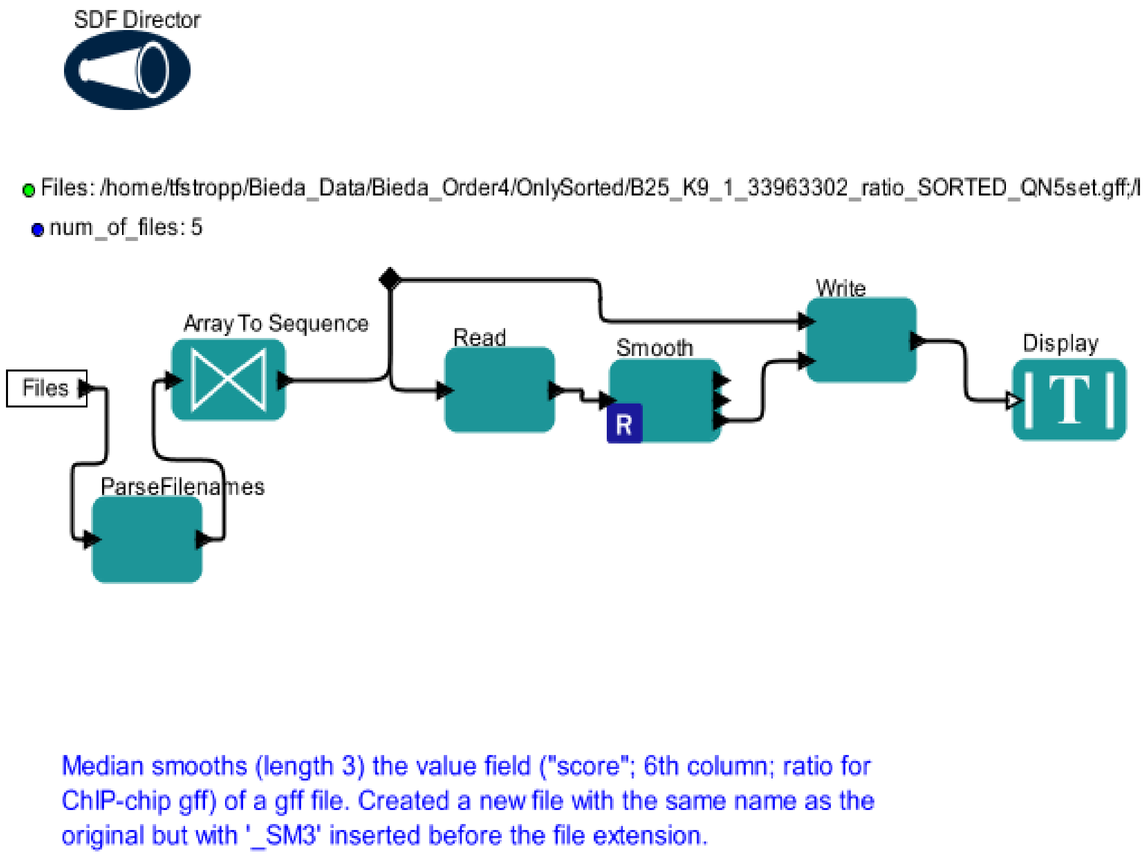

Note that “Files” exceeds the edge of this figure because there are multiple files listed. An alternative formulation would have reading of all files in a directory (see gffQN_SM3_TINY.xml for an example of this approach).

Fig. S11. gffSmooth.xml workflow.

gffSort.xml

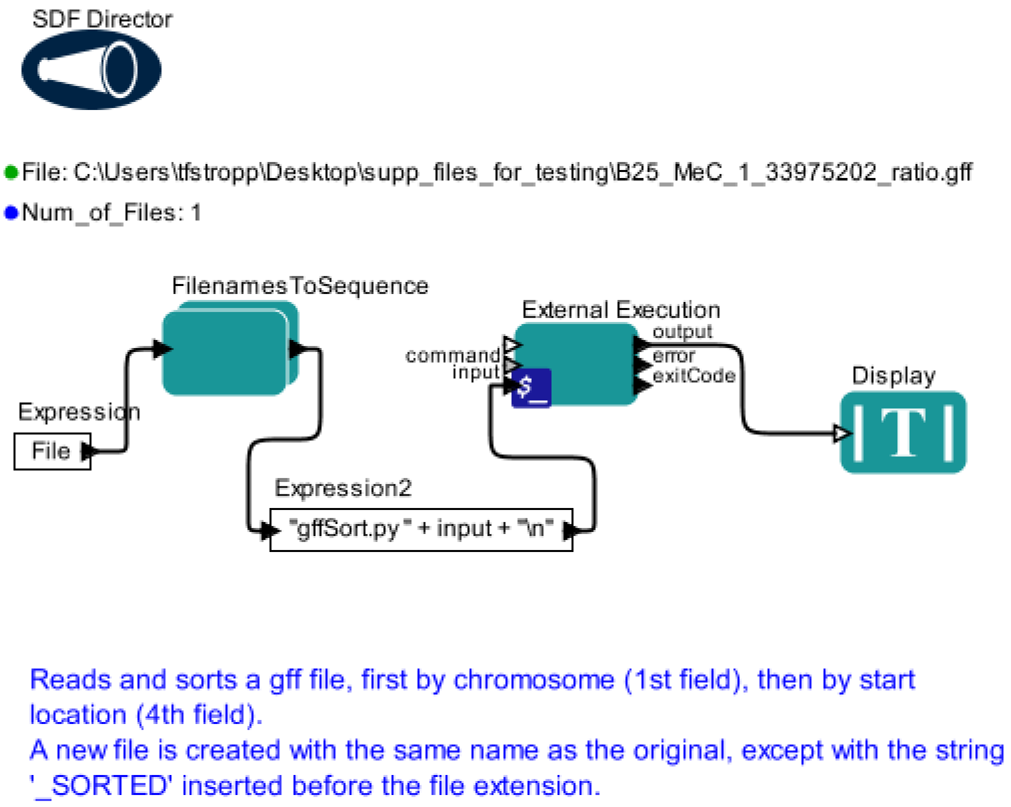

Fig. S12. gffSort.xml workflow.

gffSplit.xml

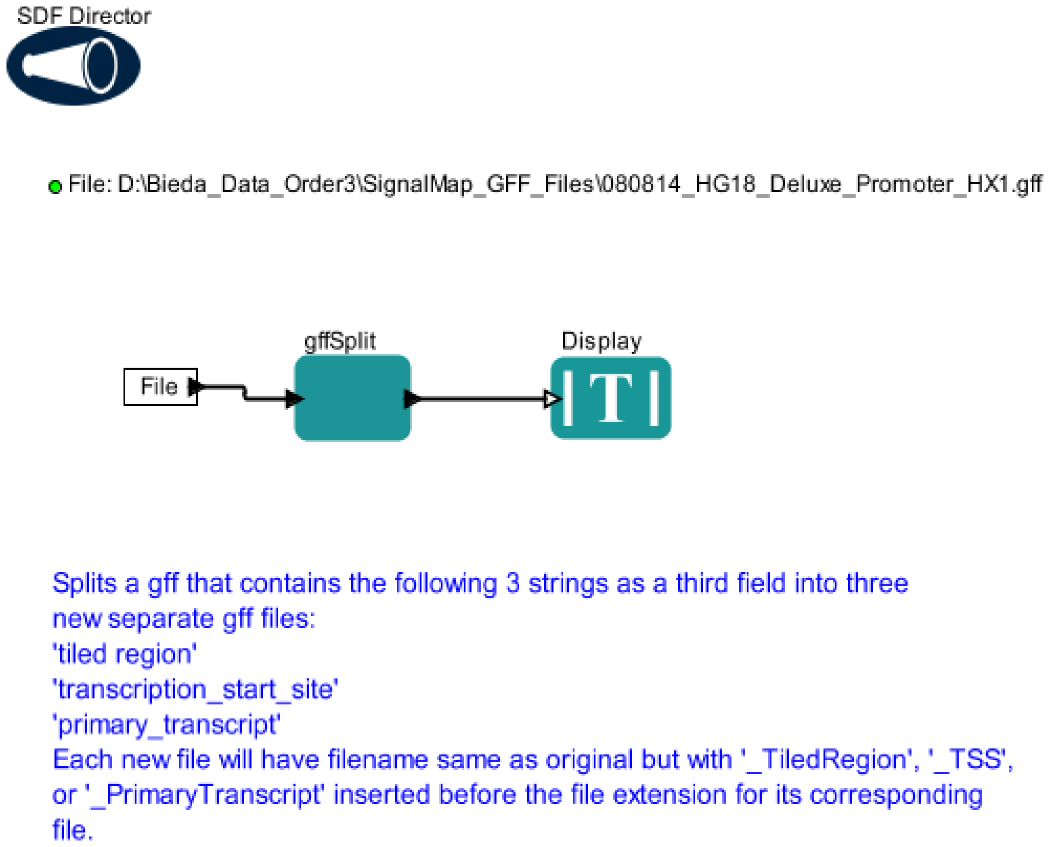

Fig. S13. gffSplit.xml workflow.

gffStats_gffRead_simple.xml

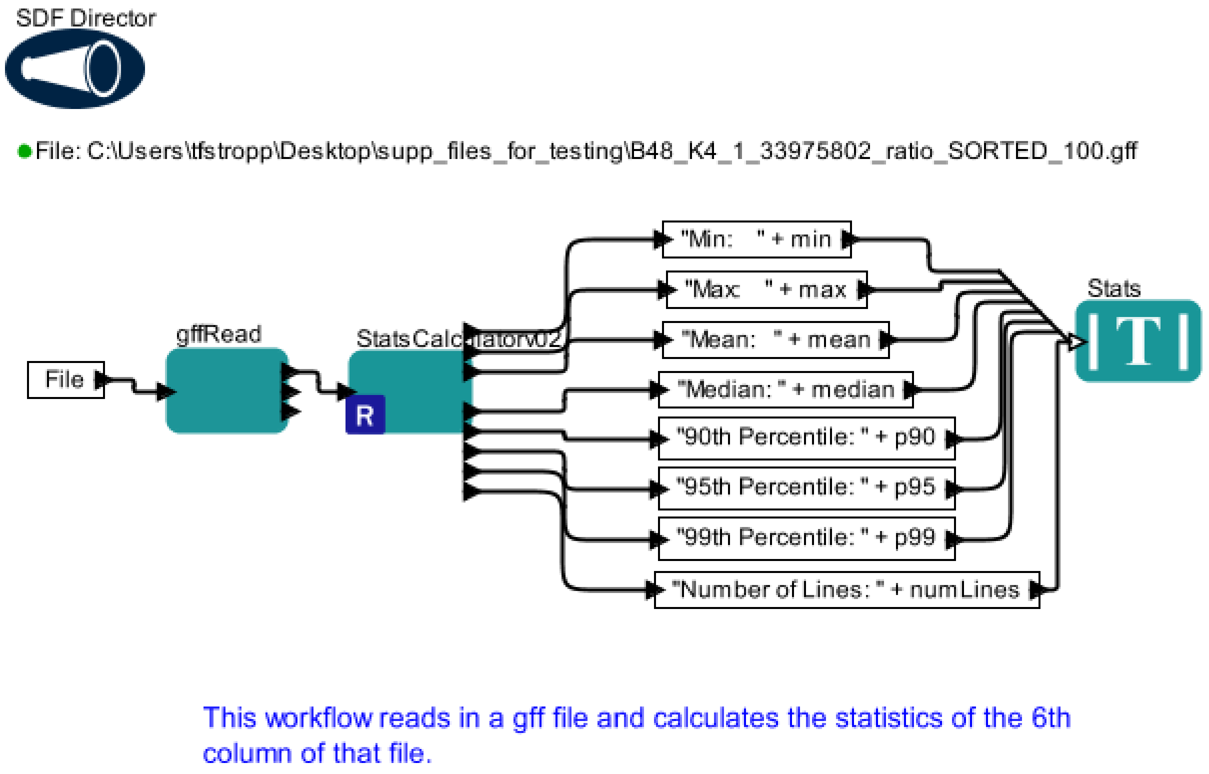

GOAL: produce statistics on the ratio column of a gff file. This is shown in Fig. 2 of Stropp et al. See results for further discussion.

Fig. S14. gffStats_gffRead_simple.xml workflow.

gffStats_Rbased_simple.xml

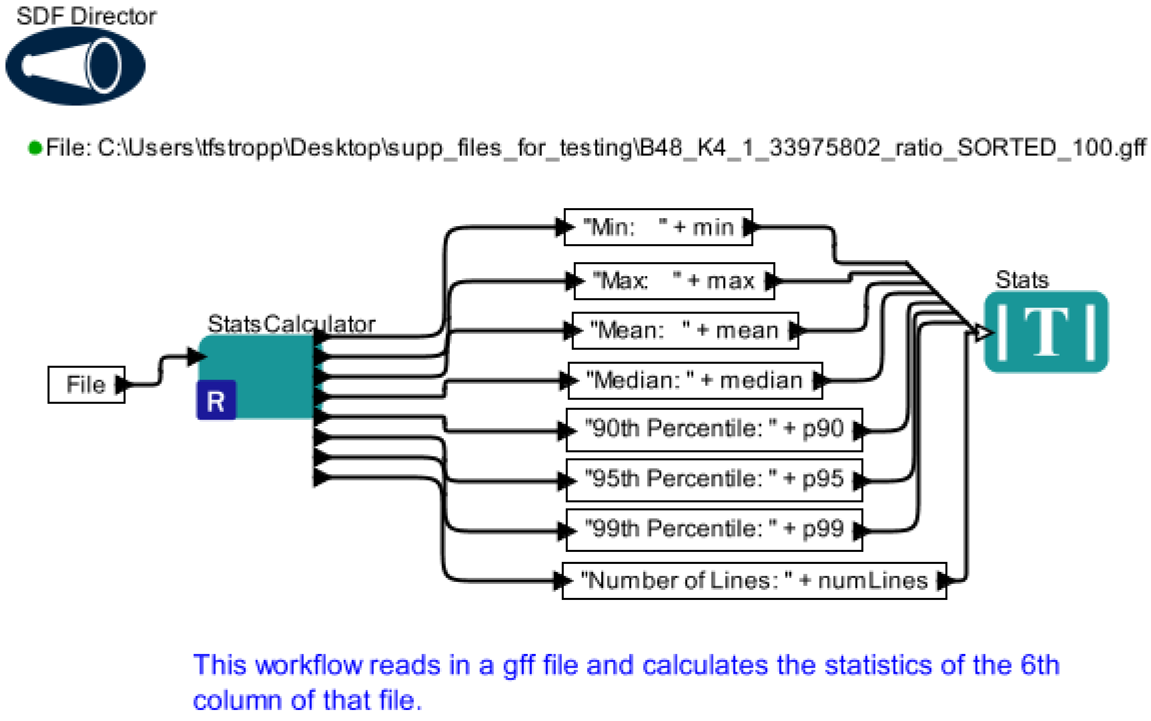

GOAL: produce statistics on the ratio column of a gff file. This is briefly discussed in RESULTS.

Fig. S15. gffStats_Rbased_simple.xml workflow.

gffSubtract.xml

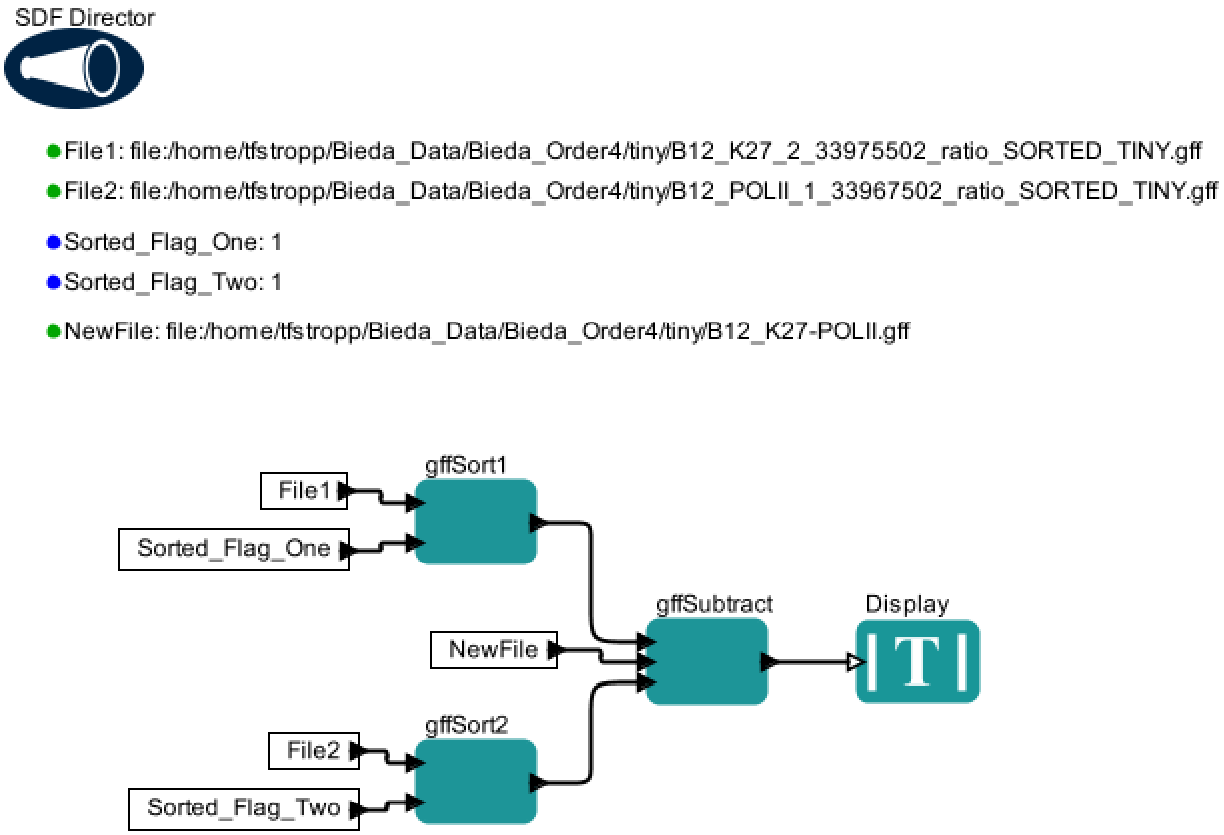

GOAL: subtract values in one GFF file from another GFF file.
Description:
File1 and File2 are the input GFF files. Critically, it is assumed that both are exactly the same format. Otherwise, subtraction may be meaningless.
NewFile is the output GFF file, which will be (File1-File2) for ratio values.
Sorted_Flag_One is an indication of whether the first file is sorted; 1 means already sorted; 0 means unsorted.
Sorted_Flag_Two is an indication of whether the first file is sorted; 1 means already sorted; 0 means unsorted.
Note that if there is doubt about sorting, both values should be set to 0. Sorting can be a slow process; these flags are a convenience to speed processing.

Fig. S16. gffSubtract.xml workflow.

PrimerDesign.xml

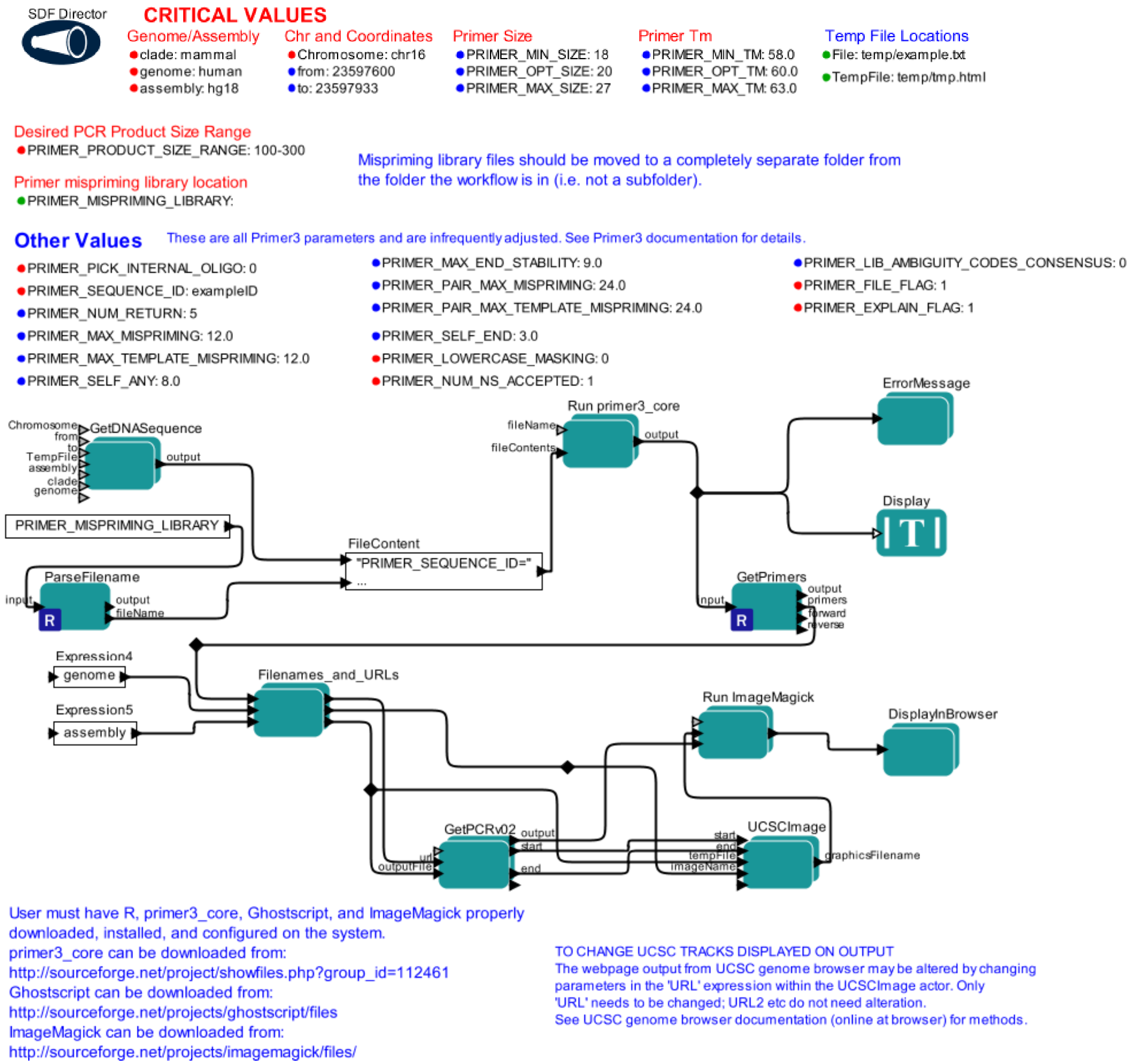

GOAL: To allow automatic design of primers. This is Figure 5 of Stropp et al. See results for full description.

DEPENDENCIES:

All packages are free open source

•Primer3 local installation

•Internet access (UCSC browser)

•ImageMagick (and GhostScript) local installation

•R

Fig. S17. PrimerDesign.xml workflow.

ProbeSpacings.xml

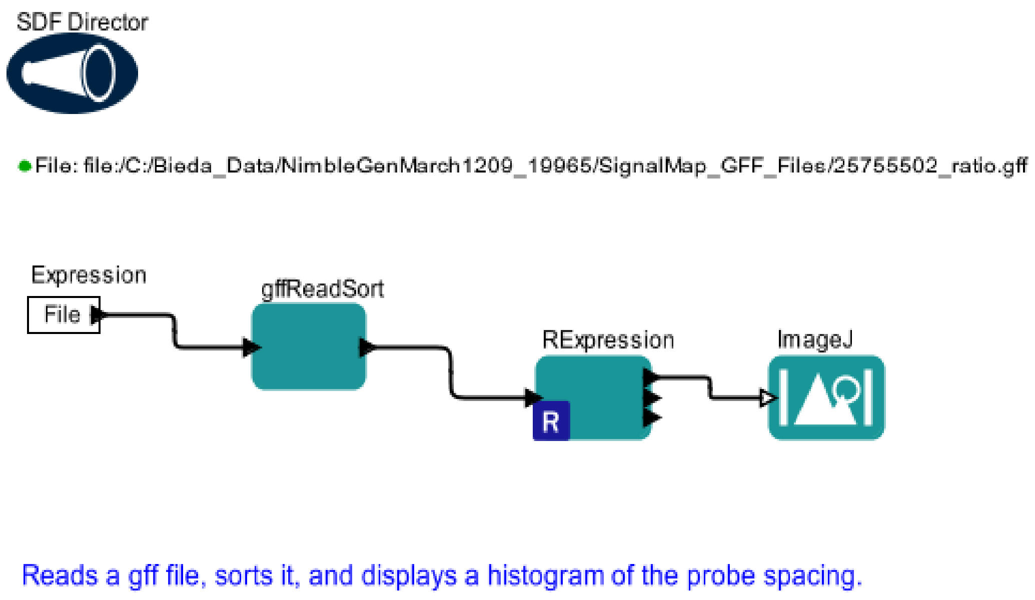

Fig. S18. ProbeSpacings.xml workflow.

QuantNorm.xml

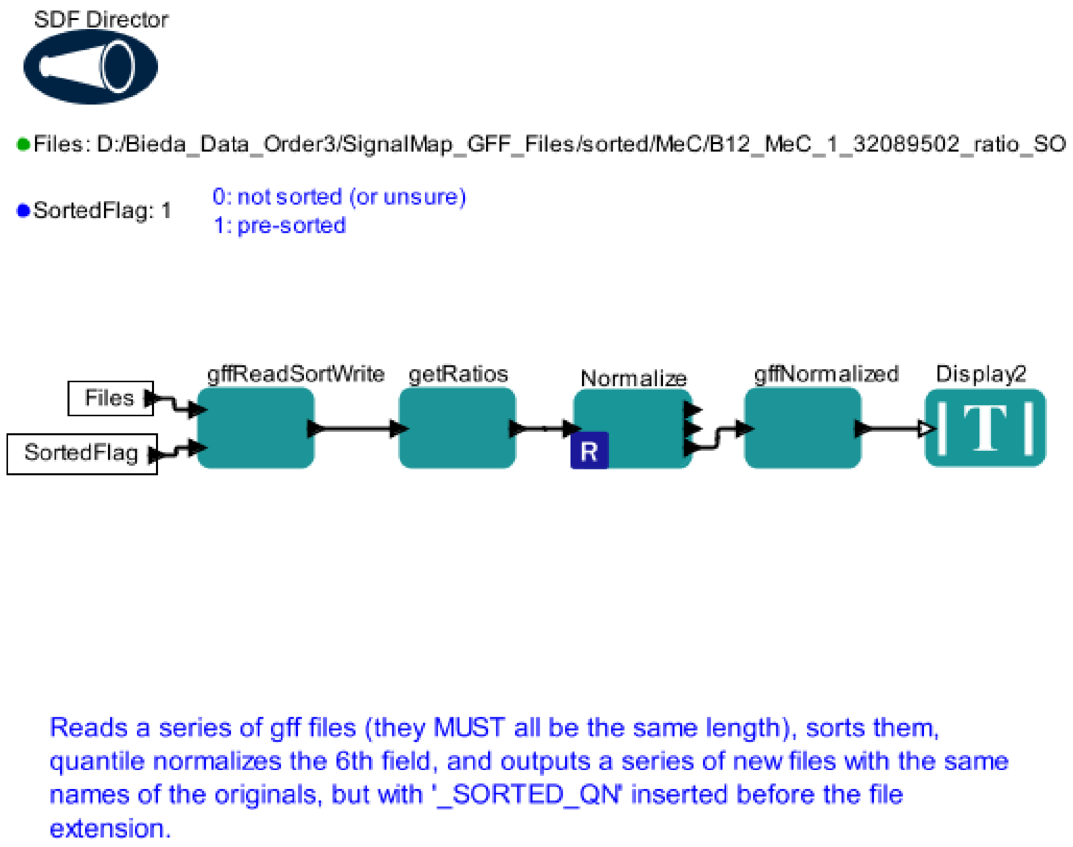

Note that “Files” exceeds the edge of this figure because there are multiple files listed. An alternative formulation would have reading of all files in a directory (see gffQN_SM3_TINY.xml for an example of this approach).

Fig. S19. QuantNorm.xml workflow.

Regex_R.xml

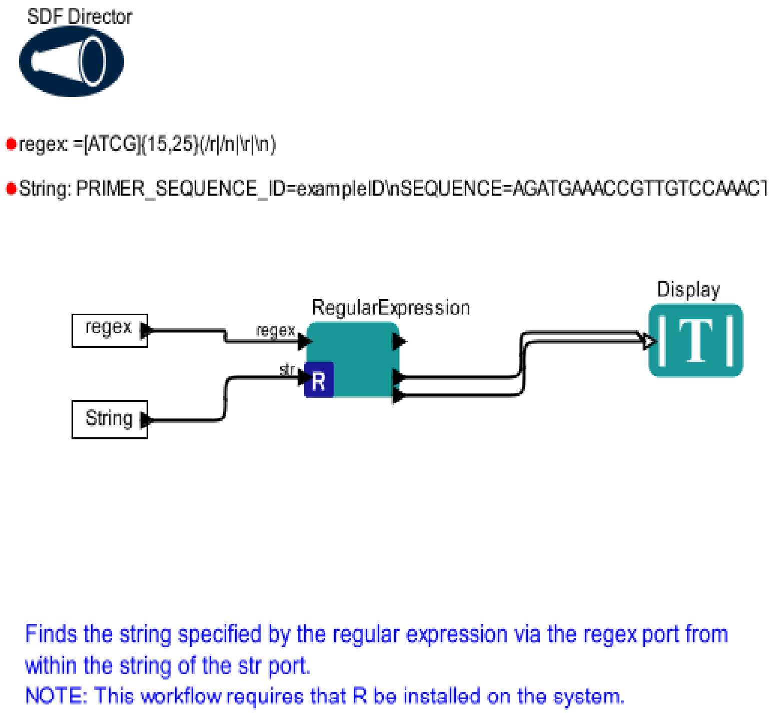

Fig. S20. Regex_R.xml workflow.

RunDetection.xml

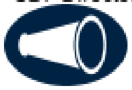

- File: file:/C:/Bieda_Data/NimbleGenMarch1209_19965/SignalMap_GFF_Files/25755502_ratio.gff
- Percentile: 75
- minRunLength: 4
- maxProbeSpacing: 1500
- Comments: Comment 01,Comment 02,Comment 03

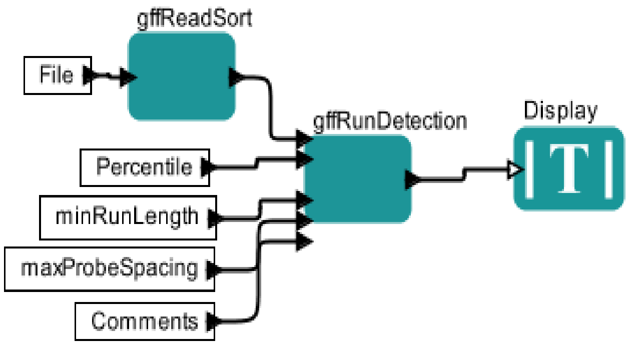

Reads a gff file, calculates the specified percentile of the 6th field, and calculates runs (of at least minRunLength, and with spacings less than maxProbeSpacing) of ratios that are greater than or equal to that value, always respecting chromosome boundaries.
It then creates a new file where the fourth field is the beginning of the run, the fifth field is the end, and the sixth field is the length.
Comments are specified via the Comments parameter and must be comma-separated. A number sign (#) will automatically be inserted at the beginning of the comment, followed by a space, and one comment will be generated per line. Comments will be inserted at the beginning of the file starting on the second line if the first line contains a comment with double number signs (##), otherwise starting on the first line.

Fig. S21. RunDetection.xml workflow.

kepler_cut.xml

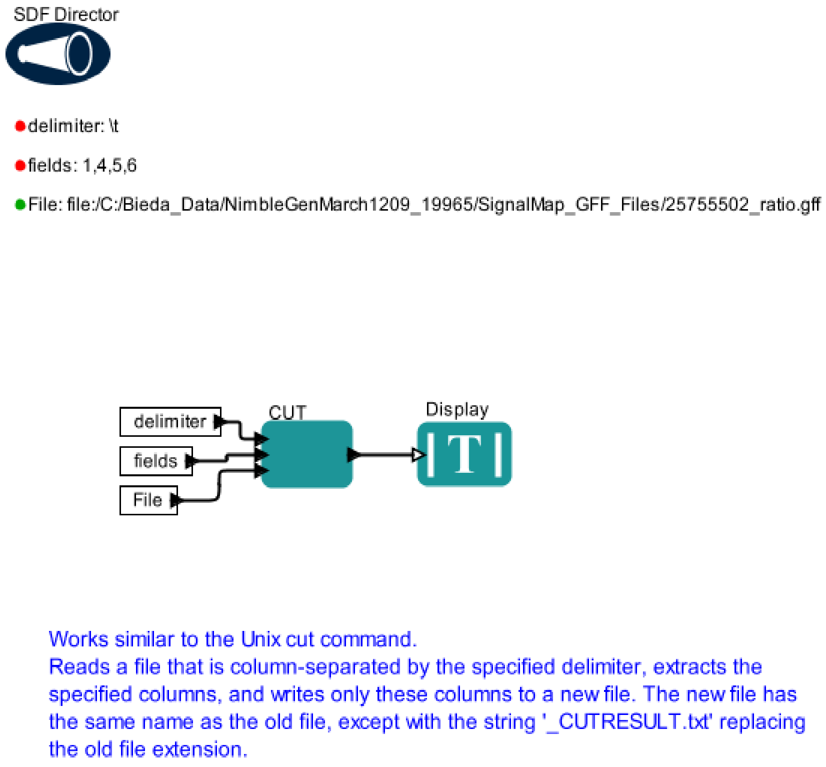

Fig. S22. kepler_cut.xml workflow.

kepler_paste.xml

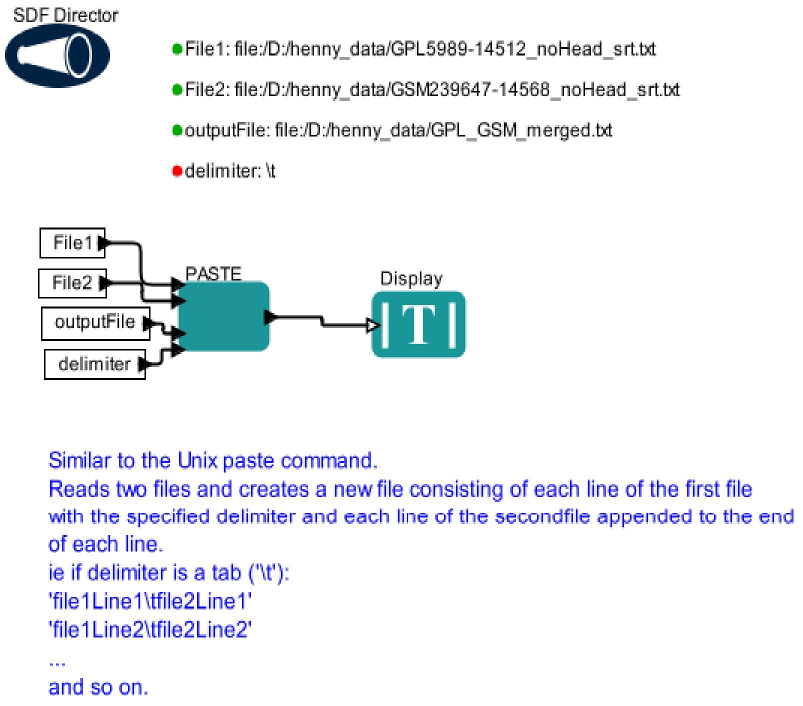

Fig. S23. kepler_paste.xml workflow.

kepler_sort.xml

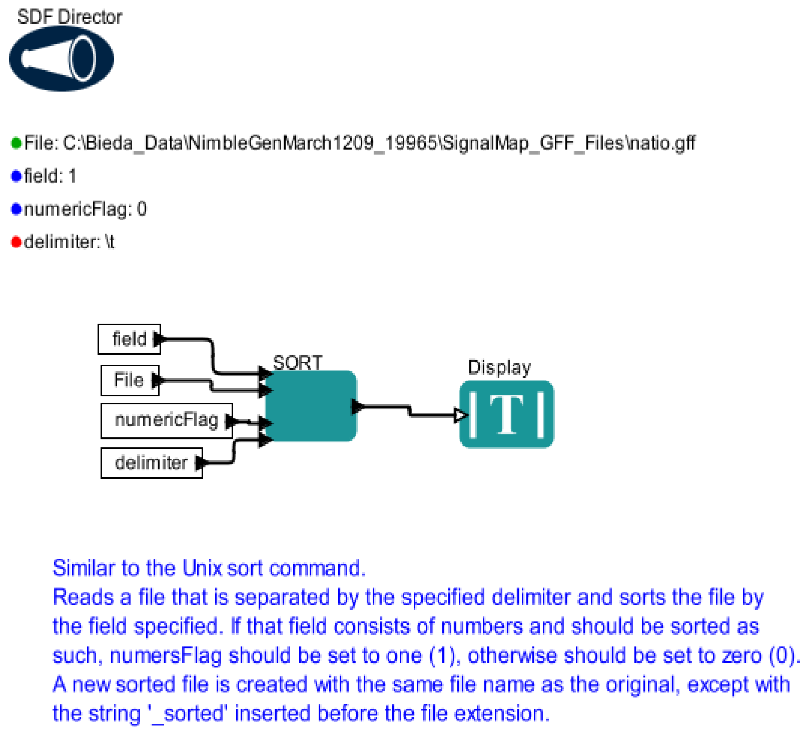

Fig. S24. kepler_sort.xml workflow.

AMDA_limmafinal.xml

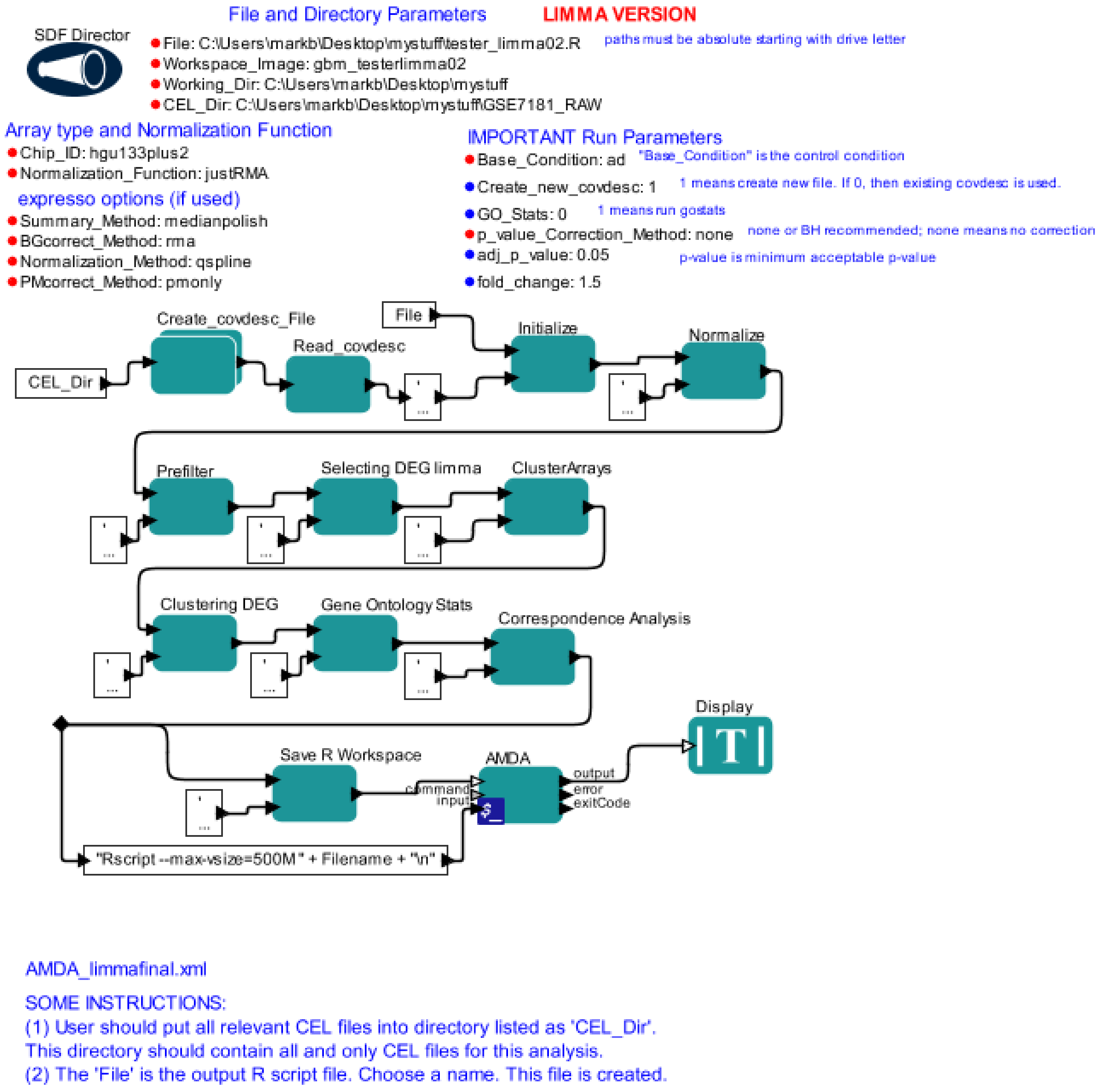

Fig. S25. AMDA_limmafinal.xml . This is a limma version of AMDA.xml. This is only a partial screenshot; there are more instructions on the actual screen.

RunDetection_with_annotation.xml

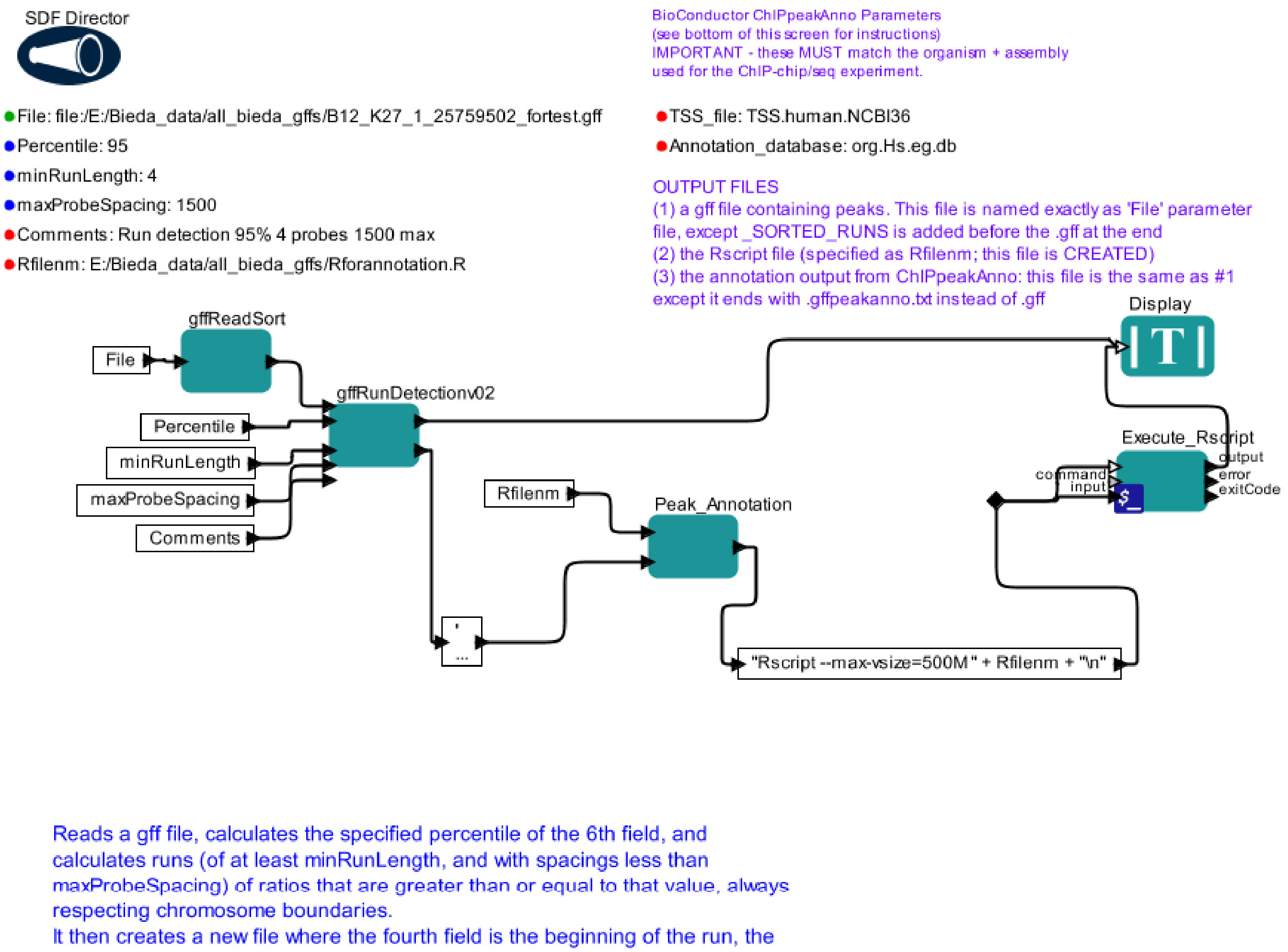

Fig. S26. RunDetection_with_annotation.xml . This is a version of the RunDetection.xml but with added annotation of peaks. Note there is more information on the actual screen than shown here; this is a slighly cropped screenshot.
