# Supplementary material for: Workflows for microarray data processing in the Kepler environment
Source: BMC Bioinformatics. 2012 May 17;13:102. doi: 10.1186/1471-2105-13-102 (PMC3431220; doi:10.1186/1471-2105-13-102)
Supplement: Additional file 2 — Stropp et al. Additional file 2: Table.pdf. This file contains Additional file 2: Table S1, which is an expanded version of Table 1 including details of implementation and operating systems among other information. Full information on installation and running workflows is listed in the “Availability” section above. [file 1471-2105-13-102-S2.pdf]

Workflow file name	Goal	inputs	outputs	Platform	speed	Notes
GFF file workflows						
AddComments.xml	Add comments to the beginning of a gff file.	filename, comments	new gff file	Vista, Linux	medium	Reads file line by line and so is not prone to memory errors, but runs slower.
DisplayRegion.xml	Create a graphical display of the value field of a GFF file (like output provided by NimbleGen SignalMap)	file, chromosome, chr_range, scale	NimbleGen SignalMap-like image	Vista, Linux	slow	primitive
GeneralHist.xml	Create a histogram of a given column of a text file. Useful for microarray gff files.	filename, column #, # of lines to skip, delimiter, xmin, xmax	a png file containing a histogram	Vista, Linux	medium	
gffFreqPoly_python.xml	Make several frequency polygons superimposed on one another for comparison. (Python version)	filenames, # of intervals, xmin, xmax, python executable filename, shell (cmd or sh, etc)	a png file of the frequency polygons of all the specified files	Vista, Linux	fast	Uses ExternalExecution actor with python program (gffRatios.py) because of speed issues.

Table S1. Listing of Workflows Developed in Kepler. These workflows are each displayed in Supplementary Figures. Table continues on further pages.

gffFullDescription.xml	Displays information about the gff file specified.	a gff filename	a couple histograms, and some text with information about the gff file	Vista	slow	relatively simple
gffMakeTinyl.xml	Greatly reduces the size of a gff so that loading and processing is much faster. Reduces file size by replacing the second, third, and last fields of the file with placeholders. Assumes that these fields are the same in all lines.	filenames, new value for third field, number of files	a new, smaller gff file	Vista	fast	**gffTiny.py** is external required program.
gffModThirdField.xml	Modify the 3rd field of a gff file.	Third field modification, position of mod, filename	a gff file with a new 3rd field	Vista, Linux	medium	Reads file line by line - slightly slower, but safer. Also adds comment to beginning of file.
gffQN_SM3_TINY.xml	Quantile Normalize, Smooth, and Tiny-ize a set of gff files. See gffMakeTiny.xml for explanation of Tiny-ize.	Directory containing files, flag if files are pre-sorted or not, flag if files should be tiny-ized, new value for 3rd field, # of files being read, string to insert before extension for new filename.	a bunch of new files that have been normalized, smoothed, and/or tiny-ized.	Vista, Linux	slow	
gffQuickLook.xml	Displays first few lines of a gff file.	gff filename, number of lines to view	the first few lines of the gff file	Vista, Linux	fast	
gffSmooth.xml	Median smooth (length 3) the 6th column of some gff files.	filenames, number of files	new gff files	Vista, Linux	medium	Reads file line by line. If 3rd field contains a different string than one of the 3 specified, those lines of the file are ignored.
gffSort.xml	Sort a gff file in chromosome + start point order (actually field 1 then field 4 order).	filenames, number of files	new sorted gff files	Vista	fast	uses external program **gffSort.py**

gffSplit.xml	Split a gff file containing the strings 'tiled region', 'transcription_start_site', and 'primary_transcript' into 3 separate files.	filename	3 new gff files	Vista, Linux	medium	Reads file line by line. If 3rd field contains a different string than one of the 3 specified, those lines of the file are ignored.
gffStats_gffread_simple.xml	Calculate min, max, mean, median, num of lines, and various percentiles of a specified field. (Python version)	Filename	Min, Max, Mean, Median, and various percentiles	Vista, Linux	slow	
gffStats_Rbased_simple.xml	Calculate min, max, mean, median, num of lines, and various percentiles of a specified field. (R version)	Filename, Column#	Min, Max, Mean, Median, and various percentiles	Vista, Linux	slow	
gffSubtract.xml	Subtract one gff file from another gff file (result based on subtraction of values in field 6).	filenames, flags if files are pre-sorted or not, new filename	new gff file	Vista, Linux	slow	
ProbeSpacings.xml	Make a histogram of the probe spacings of a gff file.	filename	a png histogram of the probe spacings	Vista, Linux	slow - medium	Reads in whole file at once. Also passes data to R actor.
QuantNorm.xml	Quantile normalize the 6th field (ratio field) of a series of gff files.	filenames, flag if files are pre-sorted or not.	new normalized gff files.	Vista, Linux	slow	Reads files line by line.
RunDetection.xml	Calculates runs of ratios (6th field) that are greater than or equal to the specified percentile of that column.	filename, percentile, minimum run length, maximum spacing between probes, comments	new gff file, where field 4 is beginning of run, 5 is end, and 6 is the run length	Vista, Linux	medium	Reads whole file at once.

RunDetection_with_Annotation.xml	Calculates runs of ratios (6th field) that are greater than or equal to the specified percentile of that column and annotates resulting peaks.	filename, percentile, minimum run length, maximum spacing between probes, comments, Rfilenm, TSS file, annotation file. These last two files are from ChIPpeakAnno BioConductor package	new gff file, where field 4 is beginning of run, 5 is end, and 6 is the run length; annotation file; Rscript file for annotation	Vista, Linux	medium-slow	Somewhat slow. Reads whole file at once.
Affymetrix Array Analysis						
AMDA.xml	Perform Affymetrix gene expression microarray analysis.	Name of R script to create, name to save R workspace, choice for working directory, object creation method, base condition, GOStats flag, directory of cell files, chip id, bg correct method, normalization method, pm corect method, summary method, ttest value, fold change value	a collection of image files and text files, the R workspace, the R script that was run	Vista	medium	REQUIRES BioConductor packages installed. See BioConductor website for instructions. Also requires package AMDA.R. See README.txt
AMDA_limmafinal.xml	Perform Affymetrix gene expression microarray analysis using limma package for DEG selection.	Name of R script to create, name to save R workspace, choice for working directory, object creation method, base condition, GOStats flag, directory of cell files, chip id, bg correct method, normalization method, pm corect method, summary method, p_value_correction method, adj_p_value, fold change value	a collection of image files and text files, the R workspace, the R script that was run	Vista	medium	REQUIRES BioConductor packages installed. See BioConductor website for instructions. Also requires package AMDA.R. See README.txt
PCR Primer Design						
PrimerDesign.xml	Pick sets of primers, given a chromosome range from user.	Large number related to primer 3 primarily; see workflow	txt file, several png files, html file showing all primer regions calculated	Vista	medium	Requires ImageMagick (and Ghostscript) and primer3_core
General Utilities						

Regex_R.xml	Simple example of find a substring within a string using regular expressions in R framework.	regular_expression, string	Array of start/end points, array of substrings found	Vista, Linux	fast	Uses R. Output format: the array of points is in the form {start1,end1,start2,end2,...} and array of substrings is {str1,str2,...}.
kepler_cut.xml	clone UNIX 'cut' command	delimiter, fields to cut, filename	new txt file containing only the cut fields	Vista, Linux	medium	partial functionality of the UNIX cut command.
kepler_paste.xml	clone UNIX 'paste' command	File1, File2, output filename, delimiter	new file containing File2 pasted to the right of File1	Vista, Linux	medium	partial functionality of the UNIX paste command.
kepler_sort.xml	clone UNIX 'sort' command	filename, field to sort, flag if data is numeric, delimiter	new file, sorted by column specified via 'field'	Vista, Linux	medium	partial functionality of the UNIX sort command.
